# Disease- and Region-Specific Molecular Patterns Involving miRNAs and Associated Signalling Pathways and Epithelial Cell-Fate Responses in PSC-Associated Ulcerative Colitis

**DOI:** 10.3390/cells15191821

**Published:** 2026-10-07

**Authors:** Alicja Łaba, Joanna Abramczyk, Michał Lubkowski, Wiesława Rogoza-Mateja, Malgorzata Milkiewicz, Oktawian Knap, Piotr Milkiewicz, Agnieszka Kempinska-Podhorodecka

**Affiliations:** 1 Department of Functional Anatomy, Pomeranian Medical University in Szczecin, Powstańców Wielkopolskich 72, 70-111 Szczecin, Poland; alicja.laba@pum.edu.pl (A.Ł.); michal.lubkowski@pum.edu.pl (M.L.); 2 Department of Medical Biology, Pomeranian Medical University in Szczecin, Powstańców Wielkopolskich 72, 70-111 Szczecin, Poland; joanna.abramczyk@pum.edu.pl (J.A.); malgorzata.milkiewicz@pum.edu.pl (M.M.); 3 Department of Gastroenterology, Pomeranian Medical University in Szczecin, Unii Lubelskiej 1, 71-252 Szczecin, Poland; wieslawa.rogoza.mateja@pum.edu.pl; 4 Department of Clinical Anatomy, Pomeranian Medical University in Szczecin, Ku Słońcu 12, 71-073 Szczecin, Poland; ok@post.pl; 5 Liver and Internal Medicine Unit, Department of General, Transplant and Liver Surgery, Medical University of Warsaw, 02-091 Warsaw, Poland; piotr.milkiewicz@wum.edu.pl; 6 Translational Medicine Group, Pomeranian Medical University in Szczecin, 70-204 Szczecin, Poland

**Keywords:** primary sclerosing cholangitis, ulcerative colitis, microRNA, miR-181, cellular fate, apoptosis, PI3K/AKT signalling, STAT3, NF-κB

## Abstract

**Highlights:**

**What are the main findings?**

- PSC-UC and conventional UC show distinct, region-specific molecular patterns involving miRNAs and associated signalling pathways, particularly miR-181a, PI3K/AKT, PDCD4, and p65.
- Regional patterns of miR-181 dysregulation differ between PSC-UC and conventional UC, while functional perturbation of miR-181a/miR-181b in colonic epithelial cells modifies cell-fate and signalling responses.

**What are the implications of the main findings?**

- These findings support the presence of disease- and region-dependent differences in molecular profiles involving miRNAs and associated signalling proteins in PSC-UC and conventional UC.
- Circulating miR-155 showed the strongest individual discrimination between PSC-UC and conventional UC (AUC = 0.797), while the exploratory multivariable miRNA profile showed high discrimination but requires independent validation.

**Abstract:**

Patients with primary sclerosing cholangitis-associated ulcerative colitis (PSC-UC) have an elevated risk of colorectal cancer compared with ulcerative colitis (UC), but the molecular mechanisms linking chronic inflammation to epithelial cell fate remain unclear. The present study investigated disease- and region-specific molecular patterns involving miRNAs and associated PI3K/AKT, STAT3, and NF-κB signalling pathways. miR-21, miR-181a, miR-181b, and key pathway components were analysed in paired ascending and sigmoid colon biopsies from patients with PSC, PSC-UC, UC, and controls. Functional effects were examined in HT-29 and NCM460D cells, and circulating miRNAs, including miR-155, were assessed. UC was characterised by marked miR-181a upregulation and PI3K/AKT activation, whereas PSC-UC showed reduced PDCD4, divergent p65 regulation, and altered miR-181a/miR-181b coordination. In NCM460D cells, miR-181a and miR-181b reduced viability, promoted apoptosis, impaired cell-cycle progression, and decreased PDCD4, p-AKT, and p-STAT3; miR-181b additionally reduced CYLD. Serum miR-155 was elevated in PSC-UC compared with UC and showed the strongest individual discriminatory capacity. Random forest analysis and an exploratory multivariable serum miRNA profile showed discriminatory potential. These findings support disease- and region-specific molecular patterns involving miRNAs and associated signalling pathways linked to epithelial cell-fate responses in PSC-UC and highlight molecular differences between PSC-UC and conventional UC.

## 1. Introduction

Primary sclerosing cholangitis (PSC) is a chronic cholestatic liver disease that is strongly associated with inflammatory bowel disease, particularly ulcerative colitis (UC) [1,2]. The distinct clinical entity of PSC-associated UC (PSC-UC) is characterised by extensive, frequently right-sided colitis and an elevated risk of colorectal neoplasia in comparison with UC alone [3]. Recent transcriptomic and multiomic studies further support molecular differences between PSC-associated disease and conventional UC, identifying distinct gene-expression profiles and, in colonic mucosal biopsies, PSC-UC-specific transcriptional and epigenetic alterations [4,5]. Chronic intestinal inflammation has been demonstrated to promote carcinogenesis through interconnected pathways that control epithelial proliferation, apoptosis, immune responses, genomic stability, and metabolism [6,7,8,9,10,11,12]. Among these, PI3K/AKT, STAT3, and NF-κB represent major signalling hubs that link inflammation to epithelial cell fate and malignant transformation [6,10,11,12]. The ability of microRNAs (miRNAs) to regulate multiple genes and pathways in a coordinated manner [13,14,15,16] suggests that their dysregulation may contribute to alterations in these signalling pathways. As was evidenced in our preceding studies, alterations involving the epithelial barrier function, bicarbonate secretion, sphingolipid metabolism, DNA mismatch repair, and immune signalling in PSC-associated colitis have been shown to be related to microRNAs [13,17,18,19]. These findings further support the presence of disease-associated molecular alterations involving multiple miRNAs and signalling pathways.

Among the microRNAs implicated in intestinal inflammation and colorectal carcinogenesis, miR-21, miR-155, and the miR-181 family are of particular relevance because they converge on PI3K/AKT, STAT3, and NF-κB signalling [20,21,22,23,24,25]. miR-21 regulates PTEN and PDCD4 [6,7,26], miR-155 contributes to SOCS1/STAT3 and DNA mismatch-repair regulation [13,26,27,28], and miR-181b can target CYLD and modulate NF-κB-related signalling [29,30]. Consequently, these microRNAs may contribute to signalling alterations associated with persistent inflammation and epithelial cell-fate responses.

However, it remains unclear whether these microRNAs and associated pathways are independently dysregulated or undergo coordinated, region-specific remodelling in PSC-associated ulcerative colitis. It was therefore hypothesized that PSC-UC is characterised by disease- and intestinal location-specific molecular alterations involving microRNAs and associated signalling pathways, which may contribute to epithelial cell-fate responses. In a preceding study, it was demonstrated that region-specific colonic miR-155 dysregulation was associated with SOCS1/STAT3 and mismatch-repair pathways in PSC and PSC-UC [13].

In order to test this hypothesis, an analysis was conducted of the colonic expression of miR-21, miR-181a, and miR-181b in combination with key components of the PTEN/AKT, PDCD4, STAT3, and CYLD/NF-κB pathways in the ascending and sigmoid colon of patients with PSC-UC, UC, PSC, and controls. A selection of microRNAs were examined in intestinal epithelial cell models to determine their effects on viability, apoptosis, cell-cycle progression, and signalling protein expression. Furthermore, circulating levels of miRNAs including miR-21, miR-155, miR-181a and miR-181b were analysed in serum. miR-155 was selected as a candidate based on hypothesis-driven findings from previous studies of tissue samples [13].

## 2. Materials and Methods

### 2.1. Study Population

The study population comprised a total of 62 participants, including 22 subjects in the control group, 12 patients diagnosed with isolated primary sclerosing cholangitis (PSC), 18 patients with PSC-associated ulcerative colitis (PSC-UC), and 10 patients with conventional ulcerative colitis (UC). The sample sizes for each assay were determined based on the availability of biological materials and are documented in Supplementary Table S1.

The inclusion criteria for patients with PSC comprised fulfilment of the EASL diagnostic criteria and an age of 18–75 years. Patients with isolated PSC had no history of concomitant inflammatory bowel disease, whereas PSC-UC was identified based on macroscopic features of UC at colonoscopy and confirmed by histological examination. Patients with UC had active disease, while control subjects underwent colonoscopy for indications unrelated to IBD and showed neither macroscopic nor microscopic abnormalities of the colon.

All patients with PSC received ursodeoxycholic acid (UDCA) at a dose of 15 mg/kg/day. Patients with PSC-UC additionally received 5-aminosalicylic acid (5-ASA), while all patients with conventional UC were also treated with 5-ASA; the daily dose of 5-ASA was 2–3 g in both groups. The main exclusion criteria included inability to provide informed consent, other chronic liver or colonic diseases, decompensated liver cirrhosis (Child–Pugh class B–C), steroid therapy, pregnancy or breastfeeding, and any condition considered by the investigators to interfere with participation or compliance.

Colon biopsies were obtained during colonoscopies from anatomically matched sites in the ascending and sigmoid colon, with three biopsy specimens collected from each region per participant. Subsequent to collection, the biopsy specimens were allocated in accordance with the downstream analysis plan. Samples intended for RNA-based analyses were placed in RNAlater RNA Stabilization Solution (Invitrogen, Thermo Fisher Scientific, Waltham, MA, USA) and subsequently stored at −80 °C until RNA isolation. Samples intended for protein analyses were snap-frozen in liquid nitrogen and stored at −80 °C until protein extraction. Samples intended for immunohistochemical analysis were fixed in formalin and subsequently processed into formalin-fixed, paraffin-embedded (FFPE) tissue blocks. Biopsy specimens from the ascending and sigmoid colon were used for tissue analyses of microRNA, mRNA, and protein, whereas serum samples were used for circulating microRNA analysis.

The study protocol was approved by the relevant Institutional Bioethics Committee (approval No. KB-0012/44/2021), and all procedures were conducted in accordance with the ethical principles of the Declaration of Helsinki. Prior to enrolment, written informed consent was obtained from all participants.

### 2.2. Cell Culture and Transient Transfection

Human colorectal adenocarcinoma HT-29 cells (ATCC^®^ HTB-38™; American Type Culture Collection, Manassas, VA, USA) and non-transformed human colonic epithelial NCM460D cells (INCELL Innovative Life Science Solutions, San Antonio, TX, USA; Cell License Material Transfer Agreement No. 204) were utilised for in vitro functional studies. The maintenance of HT-29 cells was performed in McCoy’s 5A medium (Gibco, Thermo Fisher Scientific, Waltham, MA, USA), with the addition of 10% fetal bovine serum, while NCM460D cells were cultivated in M3:10™ medium (INCELL Innovative Life Science Solutions, San Antonio, TX, USA), in accordance with the supplier’s guidelines. Cells were cultivated at 37 °C in a humidified atmosphere containing 5% CO_2_.

The employment of two distinct epithelial cell models reflected the disparate experimental inquiries addressed by the functional studies. In this study, the focus was on the examination of miR-21 in a colorectal adenocarcinoma-derived epithelial background, utilising the HT-29 cells for this purpose. Additionally, the investigation expanded to encompass the exploration of the effects of miR-181a and miR-181b in non-transformed NCM460D colonic epithelial cells. This approach was employed to assess their impact on epithelial cell fate and signalling responses in a setting that is devoid of a malignant cellular context. Consequently, the experiments were not designed for direct quantitative comparison of the effects of miR-21 with those of miR-181a or miR-181b across cell lines. The use of different cellular backgrounds should therefore be considered when interpreting the functional findings. The cells were subjected to transient transfection with a miR-21 mimic (mirVana™ miRNAs Mimic, hsa-miR-21-5p; ID MC10206), a miR-181a mimic (hsa-miR-181a-5p; ID MC10421), a miR-181b mimic (hsa-miR-181b-5p; ID MC12442), or the corresponding negative control mimic (Thermo Fisher Scientific, Waltham, MA, USA) using Lipofectamine™ RNAiMAX Transfection Reagent (Thermo Fisher Scientific), in accordance with the manufacturer’s instructions. Subsequent to this, functional and molecular analyses were performed 48 h after transfection.

The assessment of cell viability, apoptosis, and cell-cycle distribution was conducted utilising the Guava^®^ Muse^®^ Cell Analyzer (Luminex Corporation, Austin, TX, USA) and the corresponding Muse^®^ assay kits, in accordance with the manufacturer’s protocols. The expression levels of PTEN, PDCD4, phosphorylated AKT (p-AKT), phosphorylated STAT3 (p-STAT3), and CYLD were determined by Western blotting, as outlined below.

### 2.3. RNA and MicroRNA Expression Analysis

Candidate microRNAs were selected on the basis of our previous microarray profiling. miR-21, miR-181a and miR-181b were selected for tissue analyses, whereas miR-155, which had previously been characterised in PSC-associated colonic tissue [13], was additionally included as a circulating candidate driven by hypothesis.

Total RNA was isolated from serum samples using the miRNeasy Serum/Plasma Advanced Kit (Qiagen, Hilden, Germany) and from colonic tissue samples and cell pellets using the RNeasy Mini Kit (Qiagen), in accordance with the manufacturer’s instructions.

For the analysis of mRNA expression, RNA was reverse-transcribed into complementary DNA (cDNA) using the SuperScript™ IV Reverse Transcriptase Kit (Invitrogen, Thermo Fisher Scientific, Waltham, MA, USA). For the analysis of microRNA, the process of synthesising cDNA was undertaken using the TaqMan™ Advanced MicroRNA cDNA Synthesis Kit (Applied Biosystems, Thermo Fisher Scientific), in accordance with the stipulated protocol of the manufacturer.

Gene expression was quantified by real-time quantitative PCR (RT-qPCR) using TaqMan™ Gene Expression Assays. The expression levels of the following genes were analysed: PTEN (Hs02621230-m1), STAT3 (Hs00374230-m1), RELA/p65 (Hs01042014_m1) and CYLD (Hs01031576_m1). The 18S rRNA (Hs99999901_s1) was used as the endogenous reference.

miRNA expression was analysed using TaqMan™ Advanced miRNA Assays for miR-21 (477975_mir), miR-155 (477927_mir), miR-181a (477857_mir) and miR-181b (478583_mir). The endogenous control was miR-16 (477860_mir). Reactions were performed using TaqMan™ Fast Advanced Master Mix (Applied Biosystems, Thermo Fisher Scientific).

### 2.4. Western Blot Analysis

Protein lysates were prepared from colonic tissue samples and cell pellets using radioimmunoprecipitation assay (RIPA) buffer, which was supplemented with protease inhibitors and PhosSTOP™ phosphatase inhibitors (Roche, Basel, Switzerland). The total protein concentration was determined by means of the Pierce™ BCA Protein Assay Kit (Thermo Fisher Scientific, Waltham, MA, USA), in accordance with the manufacturer’s instructions. Equal amounts of total protein (30 µg per sample) were separated by SDS–polyacrylamide gel electrophoresis (SDS-PAGE) on 12% polyacrylamide gels and subsequently transferred onto polyvinylidene difluoride (PVDF) membranes.

Following the transfer process, the membranes were blocked at 4 °C for a duration of 24 h in 5% non-fat dry milk prepared in TBST. Thereafter, they were subjected to an incubation period of 2.5 h at room temperature with primary antibodies directed against PTEN (1:750; sc-7974, Santa Cruz Biotechnology, Dallas, TX, USA), CYLD (1:750; AB_2533539, Invitrogen, Thermo Fisher Scientific, Waltham, MA, USA), phosphorylated STAT3 (p-STAT3 (Tyr705); 1:500; #4113, Cell Signaling Technology, Danvers, MA, USA), PDCD4 (1:500; sc-376430, Santa Cruz Biotechnology), and phosphorylated AKT (p-AKT (Ser473); 1:500; #9271, Cell Signaling Technology). Primary antibodies were diluted in 5% non-fat dry milk in TBST.

Membranes were subsequently incubated with the appropriate horseradish peroxidase (HRP)-conjugated secondary antibodies: anti-mouse IgG (1:5000; 115-035-146, Jackson ImmunoResearch Laboratories, West Grove, PA, USA) or anti-rabbit IgG (1:5000; BA1054, Boster Biological Technology, Pleasanton, CA, USA). GAPDH (1:5000; sc-47724 HRP, Santa Cruz Biotechnology) was utilised as a loading control.

Immunoreactive bands were then subjected to visualisation using a chemiluminescent HRP substrate (Millipore, Burlington, MA, USA). The quantification of band intensities was conducted utilising the MicroChemi 2.0 imaging system in conjunction with GelQuant software (version 2.7), developed by DNR Bio-Imaging Systems, Neve Yamin, Israel. The target protein expression was then normalized to GAPDH.

### 2.5. Immunohistochemistry

Formalin-fixed, paraffin-embedded colonic tissue sections were subjected to a series of processing steps. Initially, they were deparaffinised and rehydrated. Thereafter, antigen retrieval was induced using citrate buffer (pH 6.0) at 95–100 °C for 10 min for p-STAT3, p-AKT, and p-ERK1/2, and using Tris-EDTA buffer (pH 9.0) at 95–100 °C for 10 min for Ki-67. Immunohistochemical staining was performed using primary antibodies against phosphorylated STAT3 (p-STAT3, Tyr705; 1:100; #4113, Cell Signaling Technology, Danvers, MA, USA), phosphorylated AKT (p-AKT, Ser473; 1:100; #9271, Cell Signaling Technology), phosphorylated ERK1/2 (p-ERK1/2, Thr202/Tyr204; 1:100; 28733-1-AP, Proteintech, Rosemont, IL, USA)), and Ki-67 (1:8000; 27309-1-AP, Proteintech). Primary antibodies were diluted in Phosphate Buffered Saline (PBS) containing 0.1% Tween 20.

Immunohistochemistry was performed for the purpose of representative qualitative visualisation and spatial localisation of selected signalling and proliferation markers; no quantitative scoring was undertaken.

### 2.6. Statistical Analysis

The statistical analyses were conducted utilising Statistica 13.3 (TIBCO Software Inc., Palo Alto, CA, USA) and R version 4.3.2 (R Foundation for Statistical Computing, Vienna, Austria). Continuous variables are presented as mean ± standard error of the mean (SEM), with individual observations shown where applicable. The distribution of data was evaluated through the utilisation of the Shapiro–Wilk test. Group differences were assessed using one-way analysis of variance (ANOVA) followed by Tukey’s multiple comparisons test for normally distributed data, or the Kruskal–Wallis test followed by Dunn’s multiple comparisons test for non-normally distributed data.

The effects of disease, intestinal location, and the interaction between disease and intestinal location were evaluated using a repeated measures general linear model (GLM). In this model, disease group was designated as the between-subject factor and intestinal location (ascending vs. sigmoid colon) was designated as the within-subject factor. The magnitude of the effects was expressed in terms of partial eta squared (partial η^2^). The associations between microRNA (miRNA), mRNA, and protein expression were assessed using Spearman’s rank correlation coefficient (ρ) separately within each study group and intestinal location. Principal component analysis (PCA) was utilised to evaluate the global molecular profiles, with missing values being imputed through k-nearest-neighbours imputation (kNN; k = 5).

The classification of samples as either PSC-UC or UC was performed using a Random Forest algorithm implemented in R with the ranger package (version 0.18.0). Separate models were constructed for the ascending and sigmoid colon. All 11 candidate molecular markers were entered into the Random Forest models without prior variable preselection. Each model comprised 500 trees (num.trees = 500), with three candidate variables considered at each split (mtry = 3) and a minimum terminal node size of one (min.node.size = 1). The Gini impurity criterion was utilised for the process of node splitting (splitrule = “gini”). It is important to note that these hyperparameters were not tuned, but rather were prespecified. In this context, mtry = 3 corresponds to the square-root rule for the 11 candidate molecular markers.

The performance of the model was evaluated using stratified five-fold cross-validation, a method that preserves class proportions. The calculation of the area under the curve (AUC) and the accuracy of the model were performed separately in each validation fold. The results from these calculations were then averaged across the five folds. In order to minimise information leakage, k-nearest neighbour imputation (kNN; k = 5) was performed within the training folds. The variable importance was calculated using impurity-based importance (importance = “impurity”).

Receiver operating characteristic (ROC) analysis was used to evaluate the discriminatory performance of tissue markers identified as most informative by Random Forest, individual circulating microRNAs (miRNAs), and an exploratory multivariable serum miRNA profile comprising miR-21, miR-155, miR-181a, and miR-181b, with PSC-UC defined as the positive class. AUCs with 95% confidence intervals (CIs) were calculated, and optimal cut-offs, sensitivity, and specificity were determined using Youden’s index. The multivariable miRNA profile was derived using logistic regression in the complete-case subset (*n* = 21; PSC-UC, *n* = 14; UC, *n* = 7), and paired comparisons with individual miRNAs were performed using DeLong tests in the same subjects.

Given the apparent discrimination of the multivariable serum miRNA profile, internal performance was further assessed using 2000 bootstrap resamples of the original fitted-model predictions and repeated stratified five-fold cross-validation (100 repetitions). In the latter, the logistic-regression model was refitted within each training fold and evaluated on held-out observations, with performance summarised using mean fold-specific and pooled out-of-fold area under the curve (AUCs). The logistic model indicated convergence warnings consistent with complete or quasi-complete separation, and the bootstrap procedure did not refit the model within resamples. Consequently, these analyses were interpreted with caution. The DeLong comparison between the multivariable miRNA profile and miR-181b was not interpreted because the ROC curve directions differed.

All statistical tests were two-sided, and a *p*-value of less than 0.05 was considered to be statistically significant.

## 3. Results

### 3.1. Expression of miR-21, miR-181a and miR-181b in the Ascending and Sigmoid Colon

**Figure 1:**
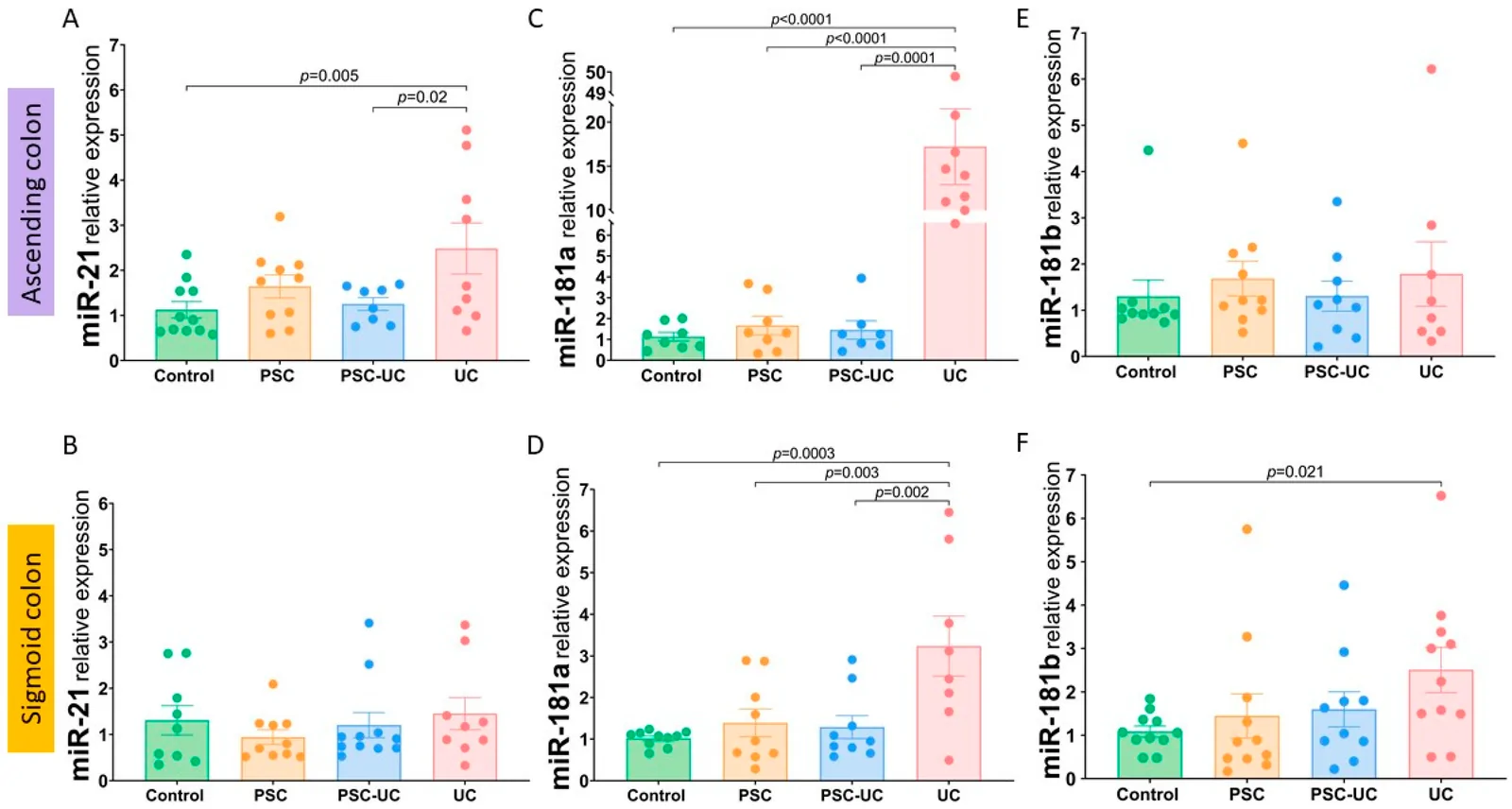
Relative expression of miR-21 (**A**,**B**), miR-181a (**C**,**D**), and miR-181b (**E**,**F**) in the ascending and sigmoid colon of control subjects and patients with PSC, PSC-UC, and UC. Relative miRNA expression was determined by RT-qPCR. Data are presented as mean ± SEM, with individual values shown. Statistical significance was assessed using one-way ANOVA followed by Tukey’s multiple comparisons test or the Kruskal–Wallis test followed by Dunn’s multiple comparisons test, as appropriate. Only statistically significant pairwise comparisons are indicated.

The expression levels of miR-21, miR-181a and miR-181b were analysed in the ascending and sigmoid colon of subjects in the control group and patients diagnosed with PSC, PSC-UC, and UC (Figure 1).

In the ascending colon, the expression of miR-21 was found to be significantly higher in patients with ulcerative colitis (2.48 ± 0.57) in comparison to controls (1.13 ± 0.18; *p* = 0.005) and patients with primary sclerosing cholangitis-ulcerative colitis (1.25 ± 0.14; *p* = 0.02) (Figure 1A). No significant differences in the expression levels of miR-21 were observed between the study groups in the sigmoid colon (Figure 1B). However, a marked increase in the expression levels of miR-181a was observed in the ascending colon of patients with UC (17.21 ± 4.30) in comparison with the control group (1.13 ± 0.21; *p* < 0.0001), PSC group (1.67 ± 0.45; *p* < 0.0001) and the PSC-UC group (1.45 ± 0.44; *p* = 0.0001) (Figure 1C).

A similar pattern was observed in the sigmoid colon, where the expression of miR-181a was significantly higher in patients with ulcerative colitis (3.23 ± 0.72) than in the control group (1.02 ± 0.06; *p* = 0.0003), in patients with primary sclerosing cholangitis (1.39 ± 0.33; *p* = 0.003) and in patients with PSC-UC (1.29 ± 0.28; *p* = 0.002) (Figure 1D).

In contrast, the expression of miR-181b differed only in the sigmoid colon, where patients with UC exhibited significantly higher expression than the control group (2.51 ± 0.52 vs. 1.09 ± 0.13; *p* = 0.021). No significant differences in the expression levels of miR-181b were observed among the remaining study groups (Figure 1E,F).

### 3.2. Expression of CYLD and p65 mRNA in the Ascending and Sigmoid Colon

**Figure 2:**
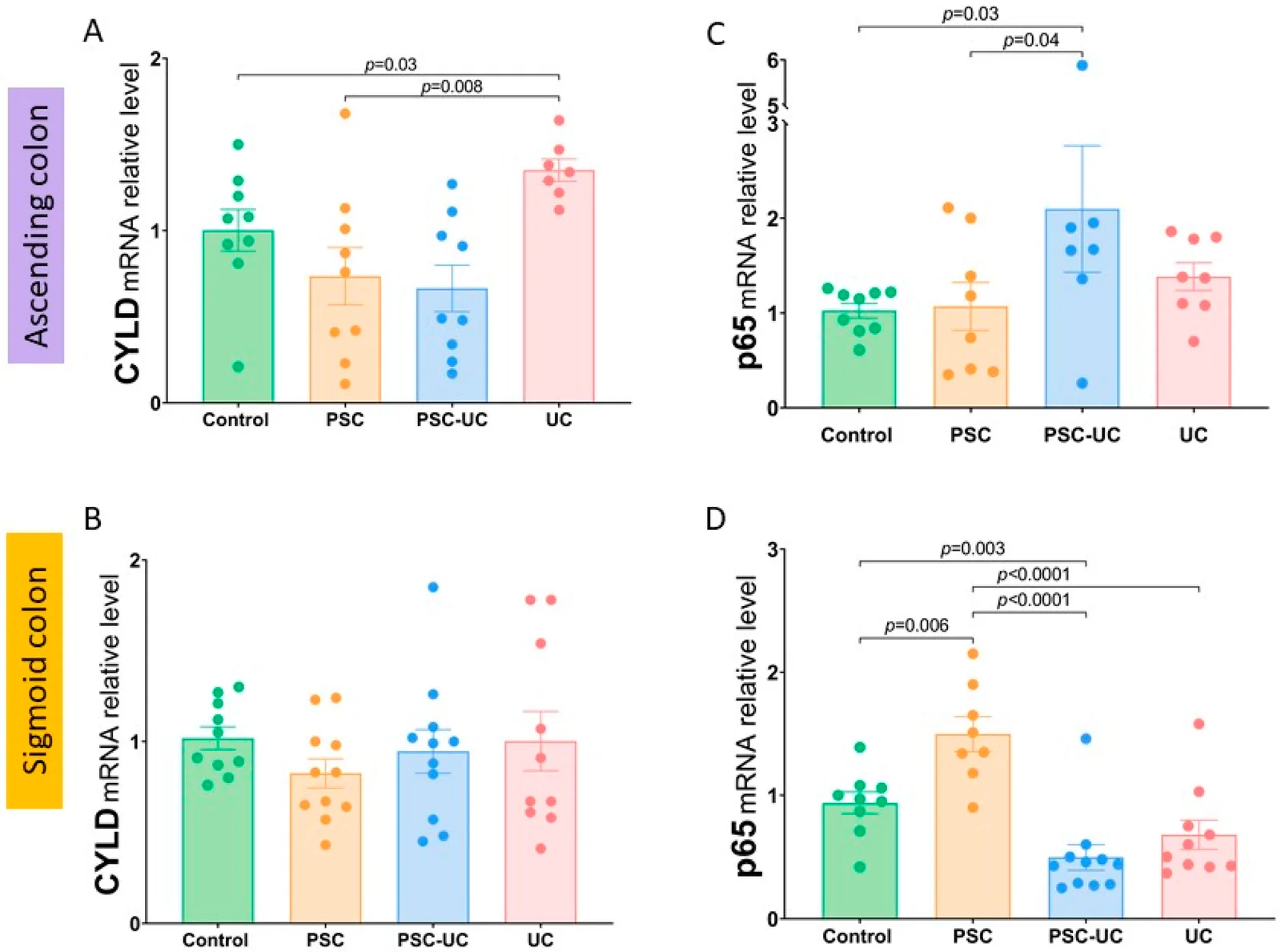
Expression of CYLD (**A**,**B**) and p65 (**C**,**D**) mRNA in the ascending and sigmoid colon of control subjects and patients with primary sclerosing cholangitis (PSC), primary sclerosing cholangitis-associated ulcerative colitis (PSC-UC), and ulcerative colitis (UC). Relative mRNA expression was determined by quantitative real-time PCR (RT-qPCR) and normalized to an endogenous reference control. Data are presented as mean ± SEM, with individual values shown. Statistical significance was assessed using one-way ANOVA followed by Tukey’s multiple comparisons test or the Kruskal–Wallis test followed by Dunn’s multiple comparisons test, as appropriate. Only statistically significant pairwise comparisons are indicated.

CYLD mRNA expression levels were found to be significantly higher in the ascending colon of patients with UC (1.35 ± 0.06) compared with the control group (1.00 ± 0.12; *p* = 0.03) and patients with PSC (0.74 ± 0.17; *p* = 0.008) (Figure 2A). No significant differences in CYLD mRNA expression were observed among the study groups in the sigmoid colon (Figure 2B).

In contrast, p65 mRNA expression exhibited a location-dependent pattern in PSC-UC. In the ascending colon, p65 mRNA expression was found to be significantly higher in patients with PSC-UC than in controls (2.10 ± 0.67 vs. 1.02 ± 0.08; *p* = 0.03) and patients with PSC (1.07 ± 0.25; *p* = 0.04) (Figure 2C). In contrast, in the sigmoid colon, p65 mRNA expression was significantly lower in patients with PSC-UC than in the control group (0.50 ± 0.10 vs. 1.00 ± 0.07; *p* = 0.003). Furthermore, patients diagnosed with PSC demonstrated significantly elevated p65 mRNA expression in the sigmoid colon (1.50 ± 0.14) in comparison to the control group (1.00 ± 0.07; *p* = 0.006), patients with PSC-UC (0.50 ± 0.10; *p* < 0.0001), and patients with UC (0.68 ± 0.12; *p* < 0.0001) (Figure 2D). No significant differences in p65 mRNA expression were detected among the remaining study groups.

### 3.3. Protein Expression

**Figure 3:**
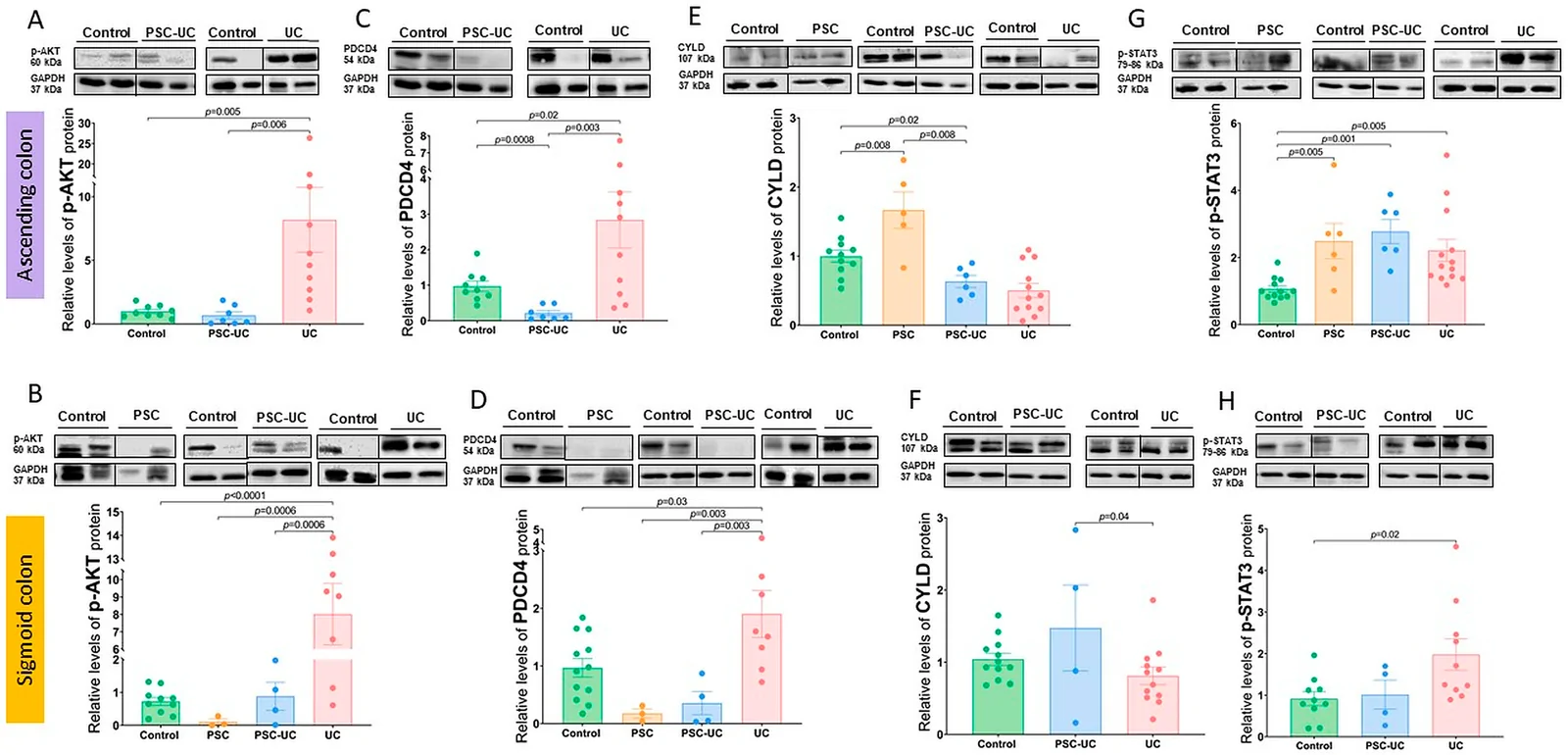
Protein expression of p-AKT (**A**,**B**), PDCD4 (**C**,**D**), CYLD (**E**,**F**), and p-STAT3 (**G**,**H**) in the ascending and sigmoid colon of control subjects and patients with primary sclerosing cholangitis (PSC), primary sclerosing cholangitis-associated ulcerative colitis (PSC-UC), and ulcerative colitis (UC). Protein expression was determined by Western blot and normalized to GAPDH expression. Data are presented as mean ± SEM, with individual values shown. Statistical significance was assessed using one-way ANOVA followed by Tukey’s multiple comparisons test or the Kruskal–Wallis test followed by Dunn’s multiple comparisons test, as appropriate. Only statistically significant pairwise comparisons are indicated.

The expression levels of p-AKT, PDCD4, CYLD, and p-STAT3 were then analysed in the ascending and sigmoid colon of subjects in the control group, as well as patients diagnosed with PSC, PSC-UC, and UC (Figure 3).

In the ascending colon, p-AKT protein expression was significantly higher in patients with UC (8.17 ± 2.55) than in controls (0.98 ± 0.16; *p* = 0.005) and patients with PSC-UC (0.67 ± 0.28; *p* = 0.006) (Figure 3A). A similar pattern was observed in the sigmoid colon, where p-AKT expression levels were significantly higher in patients with UC (8.01 ± 1.76) than in controls (0.73 ± 0.12; *p* < 0.0001), patients with PSC-UC (0.88 ± 0.42; *p* = 0.0006), and patients with PSC (0.11 ± 0.09; *p* = 0.0006) (Figure 3B).

In the ascending colon, the expression of the PDCD4 protein was found to be significantly lower in patients with PSC-UC than in the control group (0.22 ± 0.08 vs. 0.97 ± 0.14; *p* = 0.0008). In contrast, patients diagnosed with UC exhibited significantly higher PDCD4 expression (2.84 ± 0.79) in comparison to the control group (0.97 ± 0.14; *p* = 0.02) and patients diagnosed with PSC-UC (0.22 ± 0.08; *p* = 0.003) (Figure 3C). In the sigmoid colon, PDCD4 expression was also found to be significantly higher in patients with UC (1.90 ± 0.41) in comparison to controls (1.11 ± 0.16; *p* = 0.03), patients with PSC-UC (0.36 ± 0.20; *p* = 0.003), and patients with PSC (0.17 ± 0.08; *p* = 0.003) (Figure 3D).

Furthermore, CYLD protein expression was found to be significantly higher in the ascending colon of patients with PSC (1.67 ± 0.27) in comparison to the control group (1.00 ± 0.09; *p* = 0.008) and patients with PSC-UC (0.72 ± 0.12; *p* = 0.008) (Figure 3E). In the sigmoid colon, a significantly higher CYLD protein expression was observed in patients with PSC-UC (1.48 ± 0.59) compared with patients with UC (0.81 ± 0.12; *p* = 0.04) (Figure 3F).

In the ascending colon, p-STAT3 protein expression was significantly increased in all patient groups compared with the control group (1.07 ± 0.10). This increase was observed in the following patient groups: PSC (2.49 ± 0.52; *p* = 0.005), PSC-UC (2.77 ± 0.36; *p* = 0.001), and UC (2.21 ± 0.33; *p* = 0.005) (Figure 3G). In the sigmoid colon, p-STAT3 expression was significantly higher in patients with UC (1.98 ± 0.40) than in controls (0.87 ± 0.13; *p* = 0.02) (Figure 3H).

### 3.4. Disease and Intestinal Location Effects (GLM)

To distinguish the independent effects of disease status and intestinal location on marker expression, a general linear model (GLM) was applied (Table 1). Disease status significantly affected several markers, with the largest effects observed for p-AKT protein, miR-181a, and PDCD4 protein (Table 1). Intestinal location effects were observed for miR-21, miR-181a, PTEN, STAT3, p65, and p-STAT3, whereas significant disease × location interactions were identified for miR-181a, PTEN, STAT3, p65, and p-STAT3.

### 3.5. Correlation Analysis

The subsequent correlation analysis revealed disease-specific molecular relationships, with the significant associations summarised in Table 2 occurring in the sigmoid colon (Table 2; Supplementary Figure S1). In the sigmoid colon, positive PTEN–STAT3 and STAT3–CYLD correlations were demonstrated by PSC, while PSC-UC exhibited a positive miR-21–p65 correlation and a negative miR-181a–miR-181b correlation. Conversely, UC demonstrated a positive miR-181a–miR-181b correlation.

### 3.6. Principal Component Analysis

PCA demonstrated substantial overlap between PSC-UC and UC in both intestinal regions, with increased dispersion of UC samples (Supplementary Figure S2). No clear molecular separation was observed between PSC-UC and UC.

### 3.7. Machine Learning

The random forest analysis provided exploratory discrimination between PSC-UC and UC, with mean cross-validated area under the curve (AUC) values of 0.943 (accuracy 0.825) in the ascending and 0.825 (accuracy 0.756) in the sigmoid colon (Table 3). Based on impurity-based variable importance, CYLD mRNA, PTEN mRNA, miR-181a, and p-AKT were the leading variables proximally, whereas p65 mRNA, miR-21, miR-181a, and PTEN mRNA ranked highest distally. Among the individually evaluated tissue markers prioritized by Random Forest, miR-181a showed the highest AUC in the ascending colon (AUC = 0.806), whereas p65 mRNA showed the highest AUC among the evaluated markers in the sigmoid colon (AUC = 0.668) (Supplementary Table S2; Supplementary Figure S3).

### 3.8. Discriminatory Performance of Circulating miRNAs in Distinguishing PSC-UC from UC

**Figure 4:**
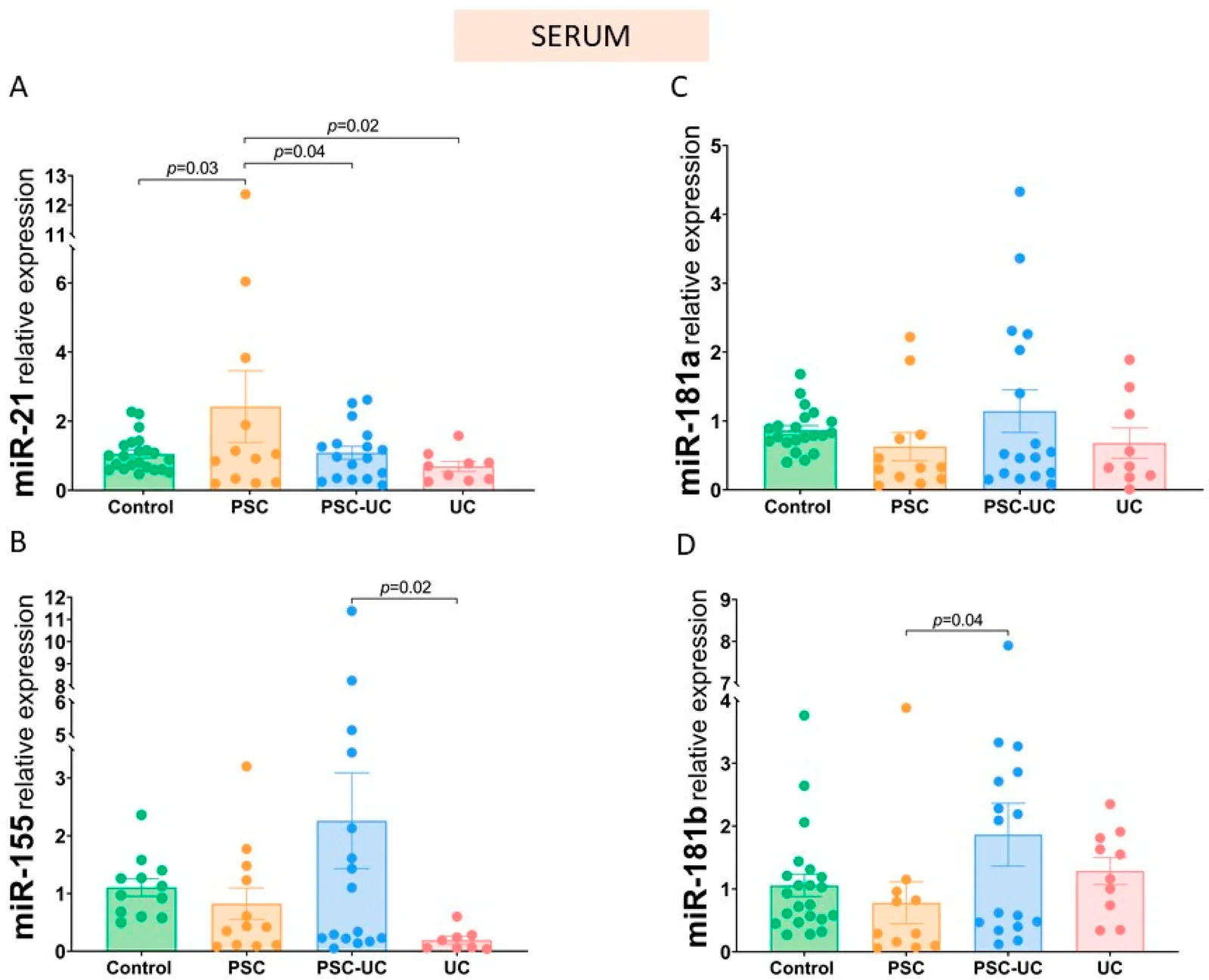
Relative expression of miR-21 (**A**), miR-155 (**B**), miR-181a (**C**), and miR-181b (**D**) in the serum of control subjects and patients with PSC, PSC-UC, and UC. Relative miRNA expression was determined by RT-qPCR. Data are presented as mean ± SEM, with individual values shown. Statistical significance was assessed using one-way ANOVA followed by Tukey’s multiple comparisons test or the Kruskal–Wallis test followed by Dunn’s multiple comparisons test, as appropriate. Only statistically significant pairwise comparisons are indicated.

The analysis of circulating microRNAs revealed differences in serum expression among the study groups. It is noteworthy that the expression of miR-155 was found to be considerably elevated in the PSC-UC group in comparison with the UC group. However, the remaining circulating miRNAs did not demonstrate any substantial differences between these two groups (Figure 4).

With the positive class defined as the PSC-UC, the highest discriminatory performance among individual circulating miRNAs was shown by miR-155 (AUC = 0.797, 95% CI: 0.613–0.981; *n*= 24), followed by miR-21 (AUC = 0.647), miR-181a (AUC = 0.582), and miR-181b (AUC = 0.547) (Supplementary Table S3; Supplementary Figure S4). The subsequent stage of the study involved an assessment of whether the combination of the four circulating miRNAs would enhance the capacity for discrimination between PSC-UC and UC.

The exploratory multivariable serum miRNA profile was evaluated in the complete-case subset of 21 subjects (PSC-UC, *n* = 14; UC, *n* = 7) and yielded an apparent AUC of 1.000. In paired analyses within the same subjects, the AUC of the multivariable miRNA profile was significantly higher than that of miR-21 (*p* = 0.00798) and miR-181a (*p* = 0.00179), whereas the difference compared with miR-155 did not reach statistical significance (*p* = 0.0582). The DeLong comparison between the multivariable miRNA profile and miR-181b was not interpreted because the ROC curve directions differed (Supplementary Table S4).

The repeated stratified five-fold cross-validation with model refitting in each training fold yielded a mean fold Area Under the Curve (AUC) of 0.951 (Standard Deviation [SD] = 0.050) and a mean pooled out-of-fold AUC of 0.933 (SD = 0.068). However, it was observed that the logistic-regression fitting generated convergence warnings that were compatible with complete or quasi-complete separation. The apparent AUC of 1.000 should therefore be interpreted with caution, given the relatively small sample size and the instability of the model. Bootstrap resampling of the original fitted-model predictions also yielded an AUC of 1.000, but because the model was not refitted within bootstrap samples, this estimate was not optimism-corrected (Supplementary Table S5).

### 3.9. Functional Effects of Selected miRNAs In Vitro

In order to investigate the functional effects of selected microRNAs implicated by the tissue analyses, HT-29 cells were subjected to transfection with a miR-21 mimic, whereas non-transformed NCM460D cells were subjected to transfection with miR-181a or miR-181b mimics.

#### 3.9.1. Effects of miR-21 Overexpression in HT-29 Cells

**Figure 5:**
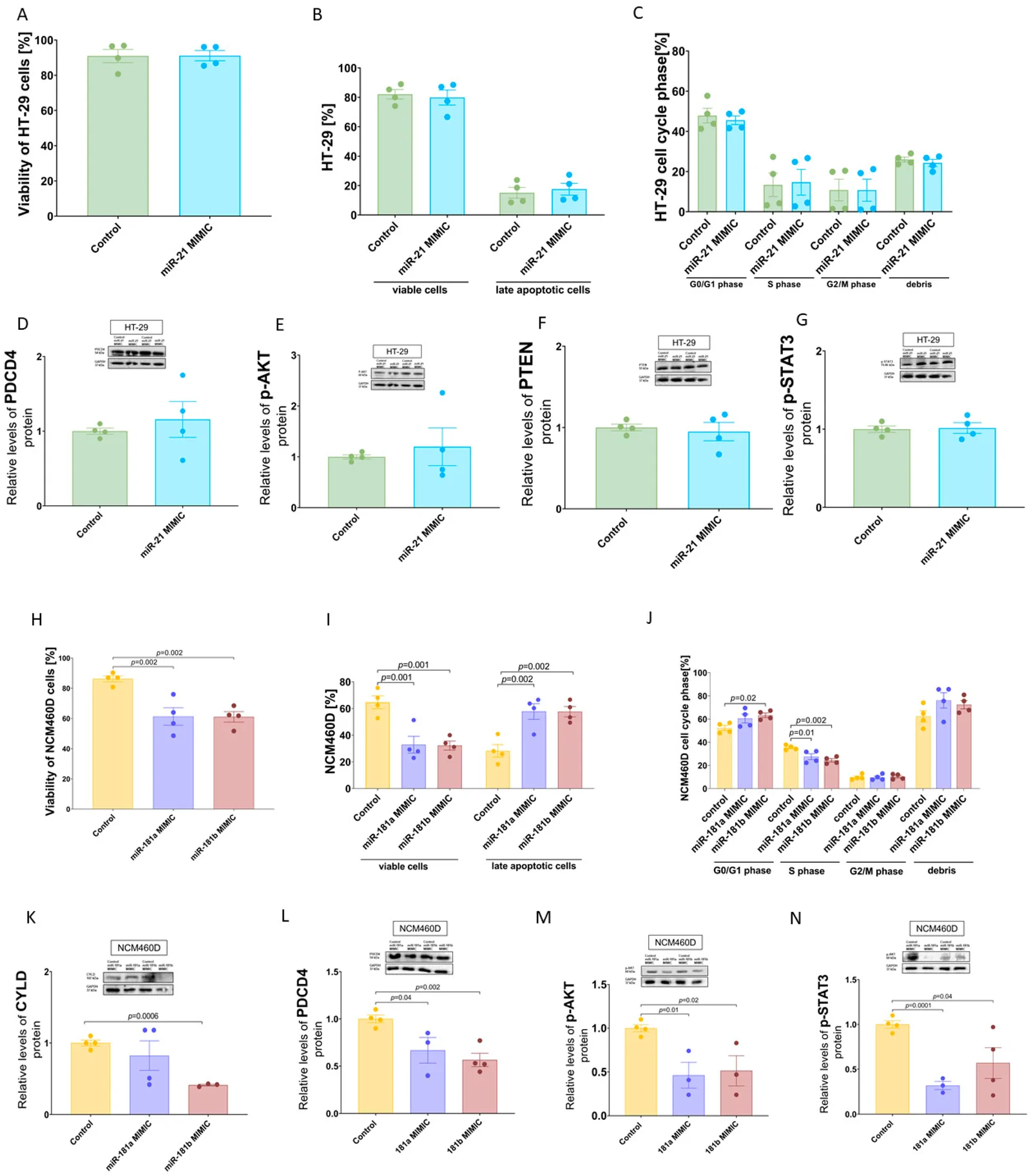
Functional effects of miR-21, miR-181a, and miR-181b overexpression in intestinal epithelial cells. Effects of miR-21 overexpression in HT-29 cells on cell viability (**A**), apoptosis (**B**), cell cycle distribution (**C**), and protein expression of p-AKT (**D**), PDCD4 (**E**), PTEN (**F**), and p-STAT3 (**G**). Effects of miR-181a and miR-181b overexpression in NCM460D cells on cell viability (**H**), apoptosis (**I**), cell cycle distribution (**J**), and protein expression of CYLD (**K**), PDCD4 (**L**), p-AKT (**M**), and p-STAT3 (**N**). Cell viability, apoptosis, and cell cycle distribution were analyzed using the Guava^®^ Muse^®^ Cell Analyzer. Protein expression was determined by Western blot and normalized to GAPDH. Data are presented as mean ± SEM, with individual values shown. Statistical significance was assessed using one-way ANOVA followed by Tukey’s multiple comparisons test or the Kruskal–Wallis test followed by Dunn’s multiple comparisons test, as appropriate. Only statistically significant pairwise comparisons are indicated.

The study found that the expression of miRNA-21 did not have a significant effect on the viability, apoptosis, cell cycle distribution, or PDCD4, PTEN, p-AKT, and p-STAT3 in comparison to control-transfected cells (Figure 5A–G).

#### 3.9.2. Effects of miR-181a and miR-181b Overexpression in NCM460D Cells

Transfection of NCM460D cells with either a miR-181a or miR-181b mimic resulted in a significant reduction in cell viability in comparison with control-transfected cells (miR-181a: 61.33 ± 5.82%, *p* = 0.002; miR-181b: 61.08 ± 3.48%, *p* = 0.002; control: 86.28 ± 1.98%) (Figure 5H).

Furthermore, both microRNAs promoted apoptosis. The proportion of viable cells was found to be significantly higher in the control group (64.71 ± 4.78%) in comparison to cells that had been subjected to transfection with the miR-181a mimic (32.86 ± 6.29%; *p* = 0.001) or the miR-181b mimic (32.20 ± 3.38%; *p* = 0.001). Conversely, the percentage of late apoptotic cells increased significantly following transfection with either the miR-181a mimic (57.75 ± 5.89%; *p* = 0.002) or the miR-181b mimic (57.61 ± 3.86%; *p* = 0.002) compared with control cells (28.24 ± 4.72%) (Figure 5I).

Cell cycle analysis demonstrated that the expression of miR-181b led to a significant increase in the proportion of cells in the G0/G1 phase (63.33 ± 3.81% vs. 52.33 ± 2.18% in controls; *p* = 0.02), accompanied by a reduction in the S-phase fraction (24.20 ± 1.47% vs. 35.40 ± 1.03%; *p* = 0.002). In a similar manner, cells transfected with the miR-181a mimic exhibited a lower proportion of cells in the S phase (27.58 ± 2.56% vs. 35.40 ± 1.03% in controls; *p* = 0.01). No significant differences were observed in the G2/M phase or in cellular debris among the study groups (Figure 5J).

Furthermore, the expression of CYLD protein was found to be significantly reduced by miR-181b (0.41 ± 0.01 vs. 1.00 ± 0.06 in controls; *p* = 0.0006), whereas miR-181a produced a similar, but less pronounced, non-significant decrease (Figure 5K).

Furthermore, both miR-181a and miR-181b have been demonstrated to significantly reduce the expression of PDCD4, phosphorylated AKT (p-AKT), and phosphorylated STAT3 (p-STAT3). The levels of the PDCD4 protein decreased following transfection with the miR-181a mimic (0.67 ± 0.14 vs. 1.00 ± 0.04 in controls; *p* = 0.04) and the miR-181b mimic (0.57 ± 0.07; *p* = 0.002) (Figure 5L).

Conversely, p-AKT expression levels were notably reduced in cells that had been transfected with the miR-181a mimic (0.46 ± 0.15; *p* = 0.01) or the miR-181b mimic (0.51 ± 0.17; *p* = 0.02) in comparison to control-transfected cells (1.00 ± 0.04) (Figure 5M). A similar reduction was observed for p-STAT3, with expression decreasing to 0.32 ± 0.05 (*p* = 0.0001) following miR-181a overexpression and to 0.57 ± 0.17 (*p* = 0.04) following miR-181b overexpression, compared with 1.00 ± 0.04 in controls (Figure 5N).

### 3.10. Immunohistochemical Localisation of Signalling Pathway Markers

Immunohistochemistry was performed to provide spatial context for selected signalling and proliferation markers within the colonic mucosa. p-AKT immunoreactivity was observed within the mucosa, including the epithelial compartment, with predominantly cytoplasmic staining (Supplementary Figure S5). The intensity and distribution of p-AKT staining appeared heterogeneous among the representative specimens and intestinal regions examined. p-STAT3 demonstrated predominantly nuclear immunoreactivity in inflammatory cells within the lamina propria, whereas epithelial cells showed weak or absent nuclear staining (Supplementary Figure S6). A comparable overall localization pattern was observed in the ascending and sigmoid colon.

p-ERK immunoreactivity was detected within the colonic mucosa, involving epithelial and lamina propria compartments (Supplementary Figure S7). The distribution and staining intensity of the samples exhibited heterogeneity, with variations observed among the representative specimens and intestinal regions.

Ki-67 exhibited nuclear immunoreactivity within colonic epithelial cells, thereby providing spatial visualisation of epithelial proliferative activity (Supplementary Figure S8). The distribution and apparent abundance of Ki-67-positive epithelial cells exhibited variation among the specimens under consideration.

As immunohistochemistry was performed solely for the purpose of representative qualitative visualisation and spatial localisation, no quantitative scoring or formal between-group statistical comparisons were undertaken.

## 4. Discussion

The present study identifies disease- and region-specific molecular patterns involving microRNAs and associated signalling pathways in PSC-UC and conventional UC. UC was characterised by marked upregulation of miR-181a and PI3K/AKT activation, whereas PSC-UC showed reduced PDCD4, divergent p65 regulation, and altered miR-181a/miR-181b coordination. Functional perturbation of miR-181a and miR-181b altered viability, apoptosis, cell-cycle progression, and PDCD4, p-AKT, and p-STAT3 expression.

Furthermore, the disease × location interactions exhibited by miR-181a, PTEN, STAT3, p65, and p-STAT3 support the contribution of intestinal location to molecular heterogeneity. This interpretation is further supported by a recent multiomic study demonstrating distinct gene-expression and DNA-methylation profiles in colonic mucosal biopsies from PSC-UC and conventional UC, including differential regulatory involvement of RELA [5]. This finding is consistent with our previous studies, which identified region-specific alterations in epithelial barrier and bile-acid-related signalling, DNA mismatch repair/SOCS1-STAT3 signalling, and immunometabolic regulation in PSC-associated colitis [13,18,19].

### 4.1. miR-21-Related PI3K/AKT and PDCD4 Signalling

UC exhibited the most pronounced PI3K/AKT activation, accompanied by elevated levels of miR-21 in the ascending colon and significantly increased p-AKT in both intestinal regions. Recent findings further support the regional relevance of miR-21 in PSC-associated intestinal disease, with increased miR-21-5p expression reported in the right colon of patients with PSC-IBD and associated with inflammatory and pro-oncogenic markers [31]. Conversely, PDCD4 levels were elevated in UC but diminished in PSC-UC. As PDCD4 is a recognised target of miR-21 [19,32], this discrepancy suggests that the observed tissue relationship between miR-21 and PDCD4 may not be indicative of a straightforward linear interaction between microRNA and target, and that additional post-transcriptional regulation and disparities in cellular composition may be present [12,32]. Furthermore, the overexpression of miR-21 did not result in a significant impact on the examined functional or signalling outcomes in HT-29 cells. In consideration of the transformed background and constitutively altered oncogenic signalling of HT-29 cells [33], it is conceivable that these negative findings may be model-dependent. The validity of this interpretation is reinforced by the existence of prior evidence demonstrating that miRNA-21 has the capacity to modulate proliferation and migration in non-transformed human colonic epithelial NCM460 cells. This observation suggests that the functional effects of this microRNA may be contingent upon the epithelial context.

### 4.2. STAT3 Signaling in the Regional Inflammatory Context

The present study found that p-STAT3 levels increased in the ascending colon in patients with PSC, PSC-UC and UC. However, the elevation of p-STAT3 was only restricted to the distal colon in patients with UC, suggesting that there is broader proximal activation of STAT3, but more disease-specific distal activation. It has been hypothesised that persistent STAT3 signalling may play a role in the inflammation-associated survival, proliferation and tumourigenesis of epithelial cells [34,35].

Immunohistochemistry further demonstrated the presence of cellular heterogeneity in the signalling process. The immunoreactivity of Phosphorylated AKT (p-AKT) was observed within the epithelial compartment (Supplementary Figure S5). In contrast, the nuclear staining of Phosphorylated STAT3 (p-STAT3) occurred predominantly in inflammatory cells within the lamina propria, with minimal or absent nuclear staining in the epithelial compartment (Supplementary Figure S6). Complementary p-ERK and Ki-67 staining provided qualitative spatial context for growth-related signaling and epithelial proliferative activity, respectively (Supplementary Figures S7 and S8). As these observations were not quantified, they do not permit formal between-group comparisons but emphasize that whole-biopsy molecular profiles may reflect contributions from different cellular compartments [34,35,36].

In conjunction with our earlier findings concerning miR-155/SOCS1/STAT3 [13], these results position STAT3 within a more extensive, region-specific network encompassing PI3K/AKT and NF-κB signalling.

### 4.3. miR-181 Family and Epithelial Cell-Fate Responses

As demonstrated in previous studies, the roles of the miR-181 family in epithelial homeostasis, inflammation, cell survival, and cancer-related signalling are context-dependent [30,37]. The present study does not therefore seek to establish microRNA-181 (miRNA-181) as a novel regulator of cell fate per se. Instead, the present study extends these observations by integrating functional miR-181 perturbation with disease- and intestinal-region-specific molecular profiles in PSC-UC and conventional UC.

The most pronounced tissue alteration was observed for miR-181a, with marked upregulation in UC in both intestinal regions, whereas miR-181b was increased only in the sigmoid colon. Neither miRNA demonstrated comparable upregulation in PSC-UC, indicating disease-specific regulation of the miR-181 family. Furthermore, the relationship between miR-181a and miR-181b exhibited divergent patterns between the two disease phenotypes, with a positive correlation observed in UC and an inverse correlation evident in PSC-UC. The findings indicate that the dysregulation of miR-181 is dependent on both the disease phenotype and the location of the intestinal tract. Consistent with CYLD being a miR-181b-responsive target [29], miR-181b overexpression significantly reduced CYLD in non-transformed NCM460D cells.

Both miR-181a and miR-181b affected multiple cellular outcomes, including reduced viability, increased apoptosis, impaired cell-cycle progression, and decreased PDCD4, p-AKT, and p-STAT3. The coexistence of reduced CYLD and increased apoptosis is not necessarily contradictory, as individual miRNAs regulate multiple targets whose combined effects determine cellular responses. CYLD should therefore be regarded as a responsive target and a potential link to NF-κB-related signalling rather than the sole mediator of the observed phenotype [29]. The discordance between tissue CYLD mRNA and protein expression further underscores the context-dependent nature of miR-181-associated molecular responses [30,37].

It is evident that patient-derived biopsies and cell-line experiments constitute distinctly disparate experimental contexts. Colonic biopsies are known to incorporate inter-individual variability, heterogeneous cellular composition, and the local inflammatory microenvironment. In contrast, cell-line experiments provide a controlled system for examining defined microRNA (miRNA) perturbations in a relatively homogeneous epithelial background. This distinction helps explain the apparent directional discordance between increased miR-181a together with increased p-AKT and p-STAT3 in UC tissue and the reduction of p-AKT and p-STAT3 following miR-181a/miR-181b overexpression in NCM460D cells. In particular, the distribution of tissue p-STAT3 revealed a predominant localization within inflammatory cells within the lamina propria, as opposed to the epithelial compartment. Consequently, parallel changes in microRNA and signalling proteins in heterogeneous tissue should not be interpreted as evidence of a direct activating relationship. The in vitro experiments demonstrate that the perturbation of miR-181a/miR-181b can modify epithelial cell fate and signalling responses. Therefore, the in vitro findings provide functional context for the molecular patterns observed in human colonic tissue rather than directly reproducing these patterns.

### 4.4. Integrated and Circulating-Marker Analyses

The PCA demonstrated substantial overlap between the PSC-UC and UC groups. In contrast, the Random Forest analysis revealed exploratory discriminatory power, particularly in the ascending colon. In view of the limited cohort size, these multivariate findings remain hypothesis-generating and require independent validation.

The circulating profiles of microRNAs exhibited significant variations compared with those observed in tissue samples. This finding is in accordance with the notion that multiple cell types and tissues contribute to the composition of circulating microRNA pools, with these pools being released through either active secretion or passive leakage [16]. Serum miR-155 levels were elevated in patients with PSC-UC compared with patients with UC alone and showed the strongest individual discriminatory performance, with an AUC of 0.797. These findings support further evaluation of circulating miR-155 as a candidate non-invasive marker [27,28].

Despite the elevated levels of circulating miR-155 observed in PSC-UC in the present study, a direct serum–tissue correlation could not be assessed due to the absence of re-measurement of miR-155 in the current tissue-expression panel. In our previous independent tissue study, which included PSC, PSC-UC, UC, and control subjects, we observed a pronounced region-specific pattern of colonic miR-155 expression. Specifically, we noted increased expression in the ascending colon of PSC and PSC-UC, but not in the sigmoid colon of PSC-UC [13]. These findings provide the biological rationale for including miR-155 as a hypothesis-driven circulating candidate in the present study. However, given the independence of the previous tissue cohort and the current serum cohort, it is not possible to infer a direct relationship between circulating and colonic miR-155. Furthermore, the availability of standardised quantitative measures of intestinal disease activity and inflammatory burden was inconsistent across the present cohort. Consequently, prospective studies incorporating paired serum and region-matched colonic tissue, in conjunction with standardised clinical and endoscopic activity assessment, will be necessary to ascertain whether circulating miR-155 reflects local colonic expression or specific PSC-UC phenotypes.

The exploratory multivariable miRNA profile showed apparent and cross-validated discriminatory potential between PSC-UC and UC; however, these findings should be interpreted cautiously given the small complete-case cohort and instability of logistic regression due to complete or quasi-complete separation.

The strengths of this study include the integrated, anatomically defined design, which combines regional tissue analyses, circulating RNA profiling, functional experiments, and the inclusion of isolated PSC. The limitations of the present study are as follows: the relatively small and assay-dependent sample sizes; the cross-sectional design; the acute models of miRNAs; and the incomplete validation of predicted interactions between miRNAs and their targets. The findings from machine learning and circulating microRNA require external validation, while whole-biopsy analyses and qualitative, non-quantified immunohistochemistry do not permit formal between-group comparisons or fully resolve cell-type-specific contributions. Although treatment exposure and the presence of active UC were documented, detailed quantitative measures of inflammatory disease activity were not available for all participants, precluding formal adjustment for differences in inflammatory burden.

## 5. Conclusions

In conclusion, PSC-associated colitis and conventional UC exhibit distinct, region-dependent molecular patterns involving miRNAs and associated signalling pathways. The UC group was characterised by increased levels of miR-181a and PI3K/AKT activation, whereas the PSC-UC group exhibited reduced PDCD4, divergent p65 regulation, and altered miR-181a/miR-181b coordination. Functional perturbation of miR-181a and miR-181b altered viability, apoptosis, cell-cycle progression, and PDCD4, p-AKT, and p-STAT3 expression. Moreover, miR-181b overexpression reduced CYLD in NCM460D cells, providing additional functional evidence that miR-181 perturbation can modify signalling responses associated with epithelial cell fate.

**Figure 6:**
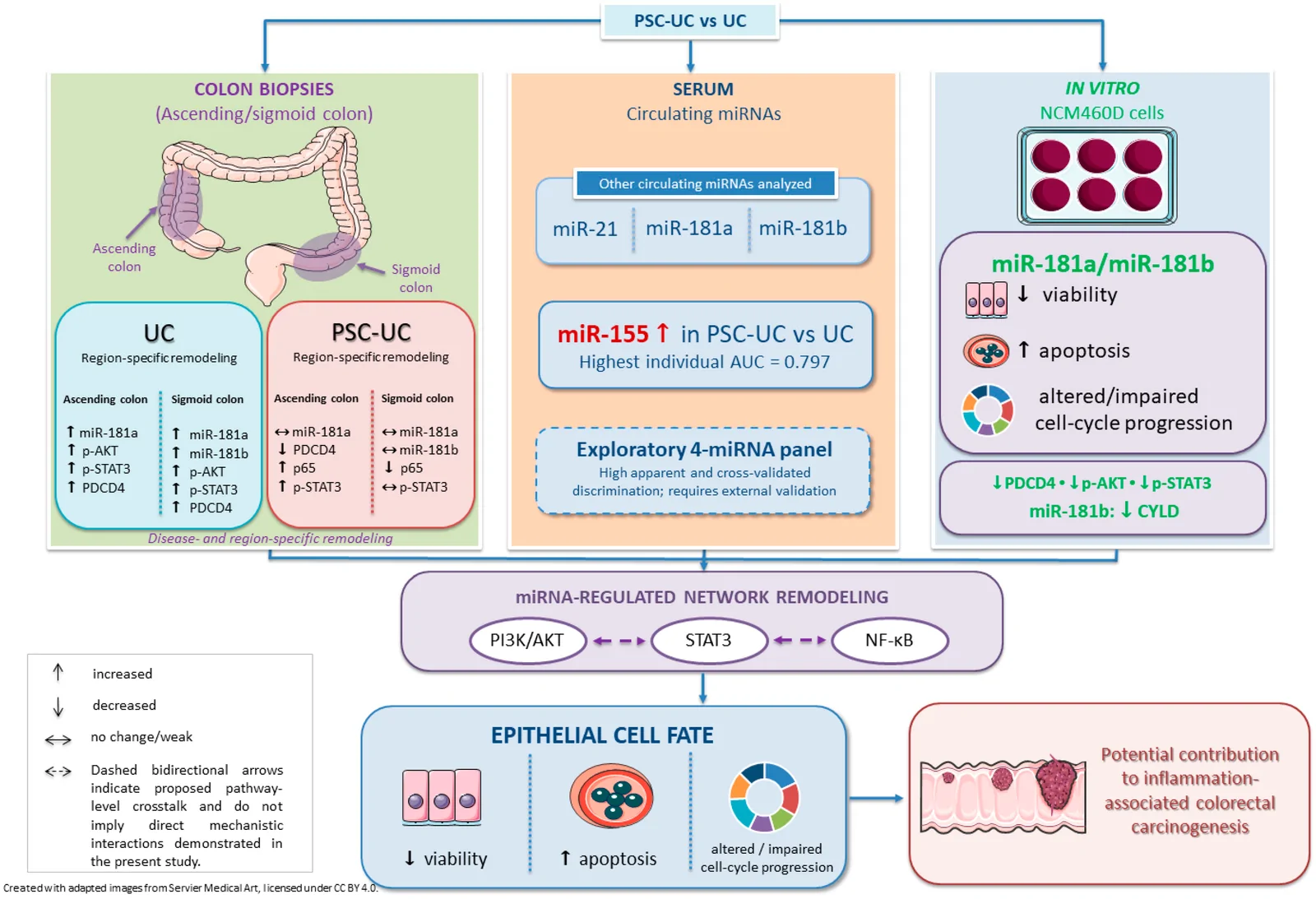
PSC-UC is characterised by disease- and intestinal location-specific molecular alterations involving microRNAs and associated signalling pathways, which may contribute to epithelial cell-fate responses.

In conjunction with our earlier research on microRNAs such as miR-346, miR-506, miR-155, and miR-125b, these findings lend support to an integrated, context- and region-dependent model of molecular alterations relevant to inflammation-associated colorectal carcinogenesis in PSC-UC. This model involves a number of processes, including epithelial defence, inflammatory signalling, genomic maintenance, bile-acid responses, sphingolipid metabolism, and cell-fate regulation (Figure 6).

## Figures and Tables

**Table 1:** Effects of disease status, intestinal location, and their interaction on marker expression determined by a general linear model (GLM).

Marker	Disease Effect (*p*, Partial η^2^)	Location Effect (*p*, Partial η^2^)	Disease × Location Interaction (*p*, Partial η^2^)
miR-21	**0.0083**, 0.162	**0.0241**, 0.075	0.297, 0.054
miR-181a	**<0.000001**, 0.420	**0.0033**, 0.140	**0.00010**, 0.302
miR-181b	0.274, 0.052	0.222, 0.020	0.841, 0.011
PTEN mRNA	0.106, 0.082	**0.00445**, 0.108	**0.00056**, 0.217
STAT3 mRNA	**0.00470**, 0.212	**0.00518**, 0.136	**0.0178**, 0.169
CYLD mRNA	**0.0151**, 0.142	0.775, 0.001	0.098, 0.088
p65 mRNA	0.080, 0.107	**0.00072**, 0.178	**0.000002**, 0.393
p-AKT protein	**0.000006**, 0.529	0.816, 0.001	0.992, 0.002
PDCD4 protein	**0.00025**, 0.388	0.352, 0.026	0.443, 0.047
CYLD protein	0.404, 0.046	0.193, 0.026	0.310, 0.082
p-STAT3 protein	**0.0157**, 0.200	**0.0290**, 0.085	**0.0163**, 0.172

Note: Bold values indicate statistically significant effects (*p* < 0.05). Partial η^2^ represents the effect size.

**Table 2:** Significant Spearman correlations between the analyzed molecular markers in the sigmoid colon of patients with PSC, PSC-UC, and UC. Only statistically significant correlations (*p* < 0.05) are presented. Spearman’s rank correlation coefficient (ρ) and corresponding *p*-values are shown.

Group	Intestinal Region	Marker 1	Marker 2	Spearman’s ρ	*p* Value
PSC	Sigmoid colon	PTEN mRNA	STAT3 mRNA	0.766	**0.0265**
	Sigmoid colon	STAT3 mRNA	CYLD mRNA	0.669	**0.0345**
PSC-UC	Sigmoid colon	miR-21	p65 mRNA	0.933	**0.000236**
	Sigmoid colon	miR-181a	miR-181b	–0.738	**0.0366**
UC	Sigmoid colon	miR-181a	miR-181b	0.833	**0.0102**

Note: Bold values indicate statistically significant *p*-values (*p* < 0.05).

**Table 3:** Impurity-based variable importance of molecular markers in Random Forest models discriminating PSC-UC from UC.

Ascending Colon Importance	Impurity-Based Importance	Sigmoid Colon	Impurity-Based Importance
CYLD mRNA	0.205	p65 mRNA	0.153
PTEN mRNA	0.166	miR-21	0.148
miR-181a	0.140	miR-181a	0.144
p-AKT protein	0.115	PTEN mRNA	0.131
CYLD protein	0.099	miR-181b	0.112
PDCD4 protein	0.076	CYLD mRNA	0.091
miR-21	0.058	STAT3 mRNA	0.082
miR-181b	0.050	PDCD4 protein	0.049
p-STAT3 protein	0.044	p-AKT protein	0.032
STAT3 mRNA	0.029	p-STAT3 protein	0.029
p65 mRNA	0.018	CYLD protein	0.028

Model performance: Variable importance was assessed using impurity-based importance. Model performance was evaluated using stratified five-fold cross-validation, with kNN imputation performed within each training fold to prevent information leakage. Mean cross-validated AUC and accuracy were 0.943 and 0.825, respectively, for the ascending colon and 0.825 and 0.756, respectively, for the sigmoid colon.

## Data Availability

The data supporting the findings of this study are currently available from the corresponding author upon reasonable request. Following publication, the data will be deposited in a publicly accessible data repository.

## Supplementary Materials

The following supporting information can be downloaded at: https://www.mdpi.com/article/10.3390/cells15191821/s1, Figure S1: Spearman correlation heatmaps of the analyzed molecular markers in the ascending and sigmoid colon of control subjects and patients with PSC, PSC-UC and UC; Figure S2: Principal component analysis (PCA) of the analyzed molecular markers in the ascending and sigmoid colon of control subjects and patients with PSC, PSC-UC and UC; Figure S3: Receiver operating characteristic (ROC) curves of the most informative molecular markers identified by Random Forest analysis for discrimination between PSC-UC and UC; Figure S4: Receiver operating characteristic (ROC) curves showing the discriminatory performance of individual circulating miRNAs (miR-21, miR-155, miR-181a, and miR-181b) for distinguishing patients with PSC-UC from those with UC; Figure S5: Representative immunohistochemical localization of phosphorylated AKT (p-AKT) in the ascending and sigmoid colon; Figure S6: Representative immunohistochemical localization of phosphorylated STAT3 (p-STAT3) in the ascending and sigmoid colon; Figure S7: Representative immunohistochemical localization of phosphorylated ERK1/2 (p-ERK1/2) in the ascending and sigmoid colon; Figure S8: Representative immunohistochemical localization of Ki-67 in the ascending and sigmoid colon; Table S1: Characteristics of the study cohorts; Table S2: Receiver operating characteristic (ROC) analysis of the molecular markers identified by Random Forest for discrimination between PSC-UC and UC in the ascending and sigmoid colon; Table S3: Discriminatory performance of individual circulating miRNAs and the exploratory multivariable serum miRNA profile for distinguishing PSC-UC from UC based on receiver operating characteristic (ROC) curve analysis. The optimal cut-off value was determined using Youden’s index; Table S4: Paired DeLong comparisons of the multivariable serum miRNA profile with individual circulating miRNAs in the common complete-case cohort (*n* = 21); Table S5: Internal performance analysis of the multivariable serum miRNA profile derived using logistic regression in the complete-case cohort (*n* = 21; PSC-UC, *n* = 14; UC, *n* = 7).

## Abbreviations

The following abbreviations are used in this manuscript:

Abbreviation	Definition
PSC	Primary sclerosing cholangitis
PSC-UC	Primary sclerosing cholangitis-associated ulcerative colitis
UC	Ulcerative colitis
miRNA	microRNA
PI3K	Phosphoinositide 3-kinase
AKT	Protein kinase B
STAT3	Signal transducer and activator of transcription 3
NF-κB	Nuclear factor kappa B
PDCD4	Programmed cell death protein 4
CYLD	CYLD lysine 63 deubiquitinase
PCA	Principal component analysis
ROC	Receiver operating characteristic
AUC	Area under the curve
GLM	General linear model
PBS	Phosphate-buffered saline
SEM	Standard error of the mean
ANOVA	Analysis of variance
kNN	k-nearest neighbours
