# Supplementary material for: Disease- and Region-Specific Molecular Patterns Involving miRNAs and Associated Signalling Pathways and Epithelial Cell-Fate Responses in PSC-Associated Ulcerative Colitis
Source: Cells. 2026 Oct 7;15(19):1821. doi: 10.3390/cells15191821 (PMC13664130; doi:10.3390/cells15191821)
Supplement: Supplementary file 1 [file cells-15-01821-s001.zip › cells-4578059-supplementary.pdf]

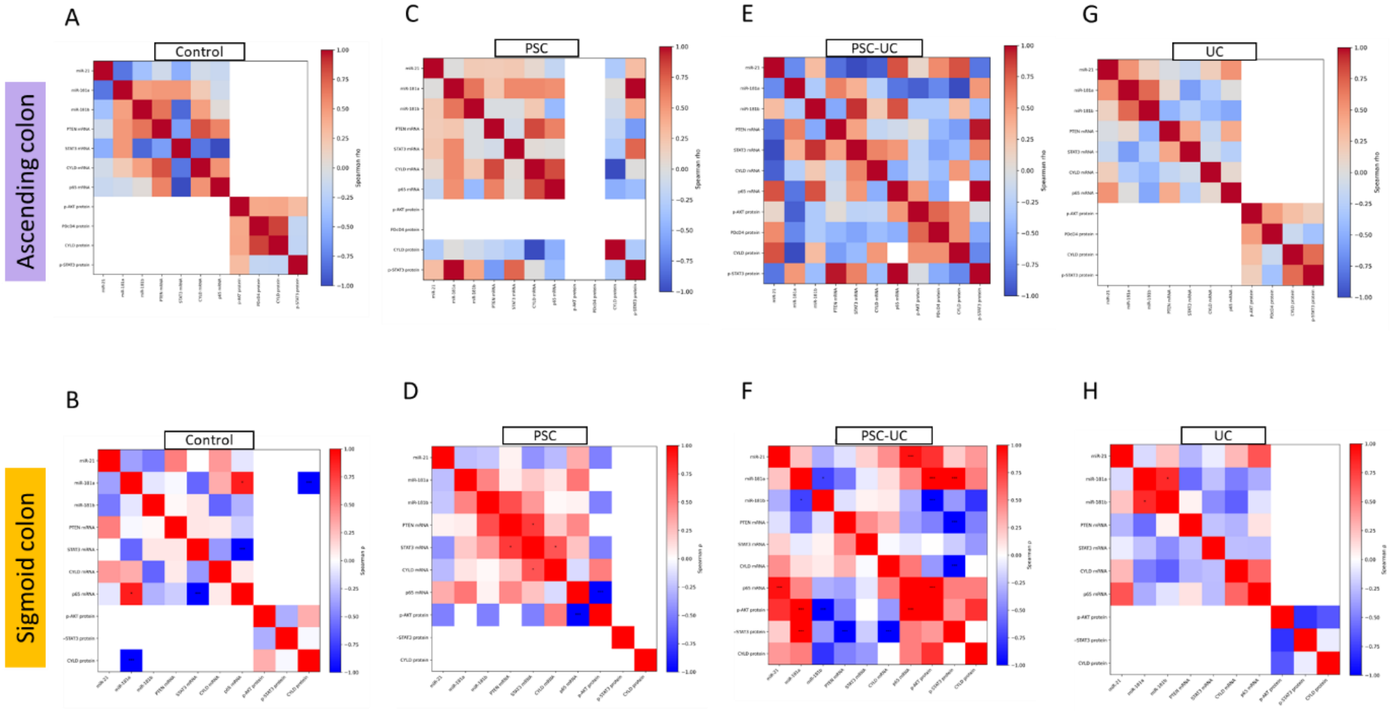

Figure S1. Spearman correlation heatmaps of the analyzed molecular markers in the ascending and sigmoid colon of control subjects and patients with PSC, PSC-UC and UC. (A,C,E,G) Ascending colon: Control, PSC, PSC-UC and UC, respectively. **(B,D,F,H)** Sigmoid colon: Control, PSC, PSC-UC and UC, respectively. Heatmaps display Spearman's rank correlation coefficients (ρ) between the analyzed miRNAs, mRNA transcripts and proteins. Red indicates positive correlations and blue indicates negative correlations, with color intensity corresponding to the strength of the correlation. Statistically significant correlations are indicated by asterisks (* $p < 0.05$, ** $p < 0.01$, *** $p < 0.001$).

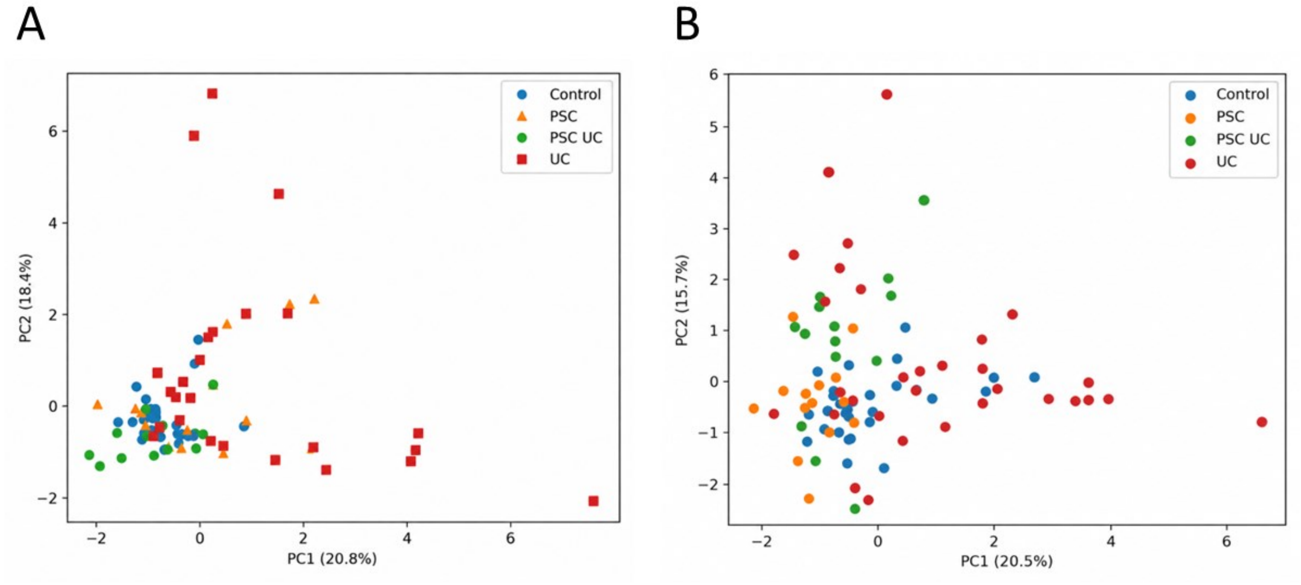

Figure S2. Principal component analysis (PCA) of the analyzed molecular markers in the ascending and sigmoid colon of control subjects and patients with PSC, PSC-UC, and UC. (A) Ascending colon. **(B)** Sigmoid colon. PCA was performed using all analyzed miRNA, mRNA, and protein markers following k-nearest neighbors (kNN, k = 5) imputation of missing values. Each point represents an individual sample, and colors indicate the study groups.

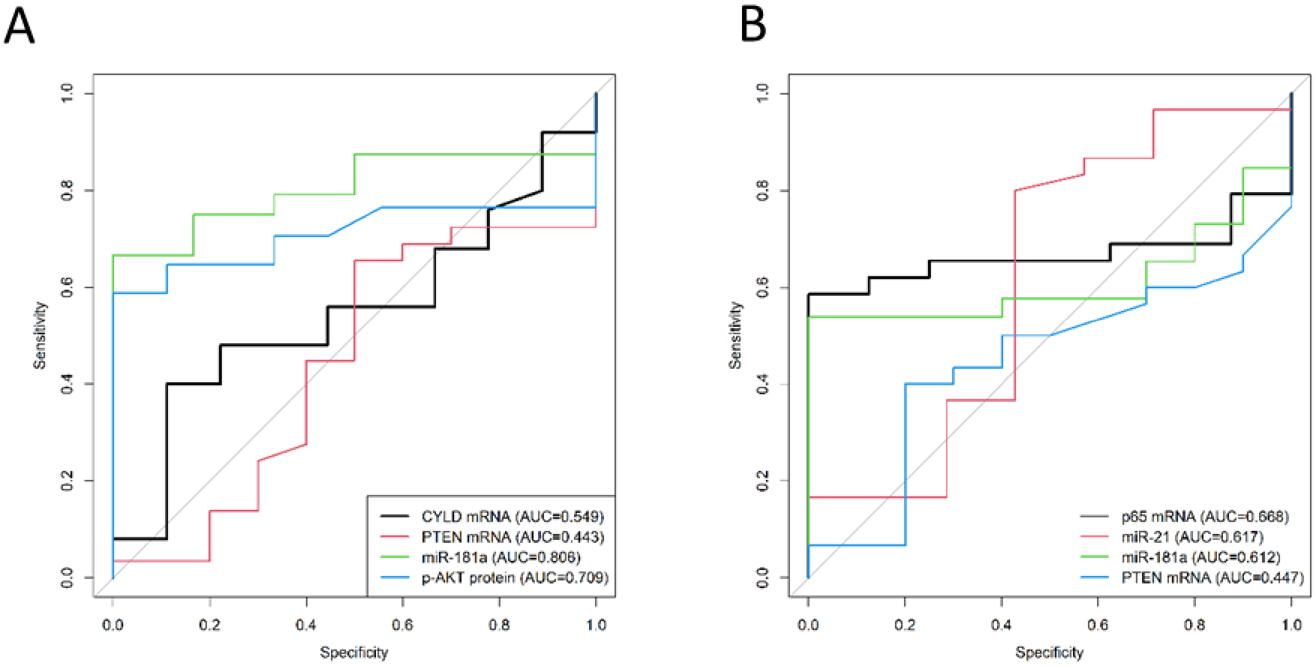

Figure S3. Receiver operating characteristic (ROC) curves of the most informative molecular markers identified by Random Forest analysis for discrimination between PSC-UC and UC. (**A**) Ascending colon, showing ROC curves for CYLD mRNA, PTEN mRNA, miR-181a and p-AKT protein. (**B**) Sigmoid colon, showing ROC curves for p65 mRNA, miR-21, miR-181a and PTEN mRNA. The gray diagonal line represents the reference line for a non-discriminatory classifier.

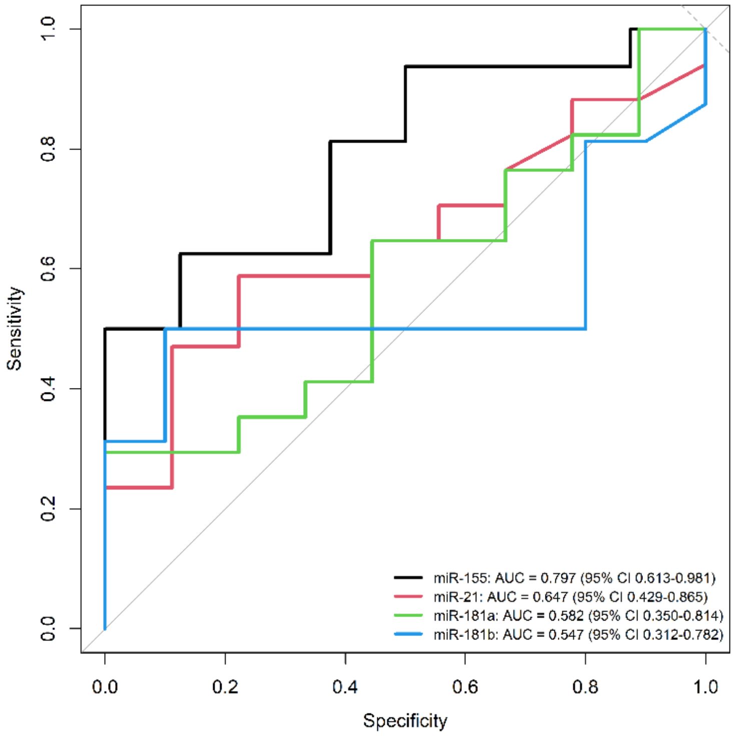

Figure S4. Receiver operating characteristic (ROC) curves showing the discriminatory performance of individual circulating miRNAs (miR-21, miR-155, miR-181a, and miR-181b) for distinguishing patients with PSC-UC from those with UC. ROC curves were generated using the original, non-inverted serum miRNA expression values. PSC-UC was defined as the positive class and UC as the negative class. Among the individual circulating miRNAs, miR-155 showed the highest discriminatory performance (AUC = 0.797).

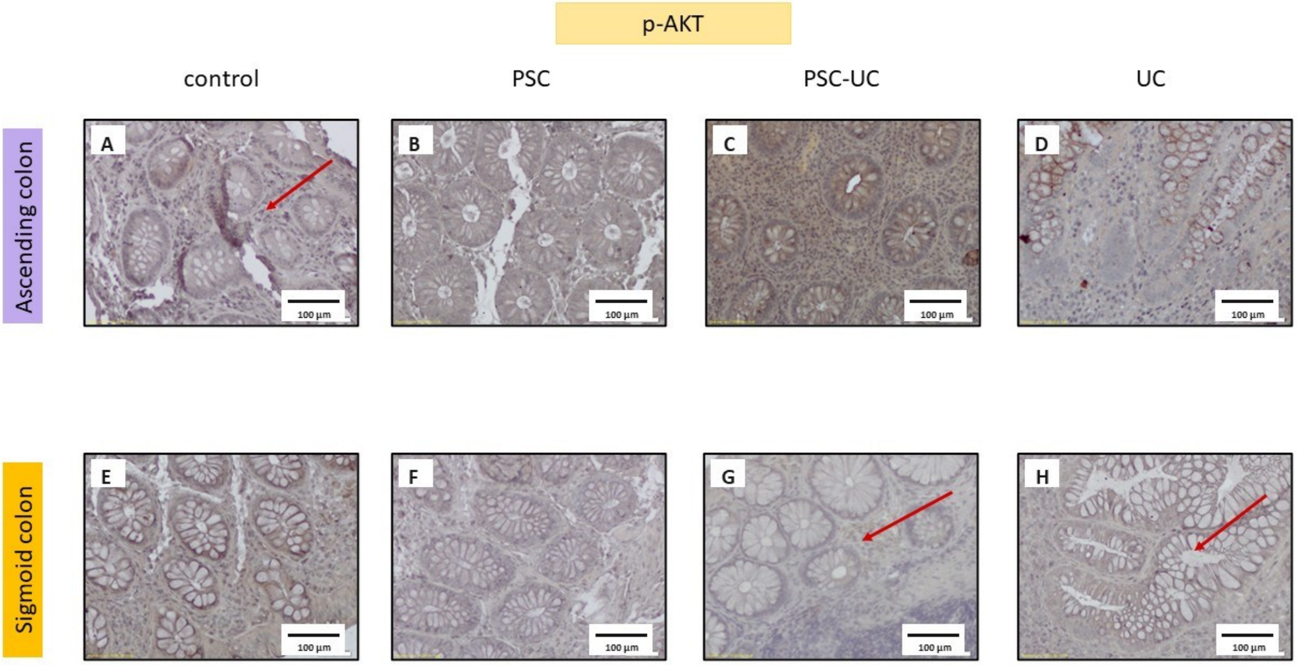

Figure S5. Representative immunohistochemical localization of phosphorylated AKT (p-AKT) in the ascending and sigmoid colon. Representative sections from control subjects and patients with primary sclerosing cholangitis (PSC), primary sclerosing cholangitis-associated ulcerative colitis (PSC-UC), and ulcerative colitis (UC) are shown. (**A–D**) Ascending colon: Control, PSC, PSC-UC, and UC, respectively. (**E–H**) Sigmoid colon: Control, PSC, PSC-UC, and UC, respectively. p-AKT immunoreactivity is detectable within the epithelial compartment, with heterogeneous staining intensity and distribution among representative specimens and intestinal regions. Images are presented as a qualitative histological illustration of p-AKT localization; no quantitative IHC scoring or between-group statistical comparisons were performed. Scale bar = 100 µm.

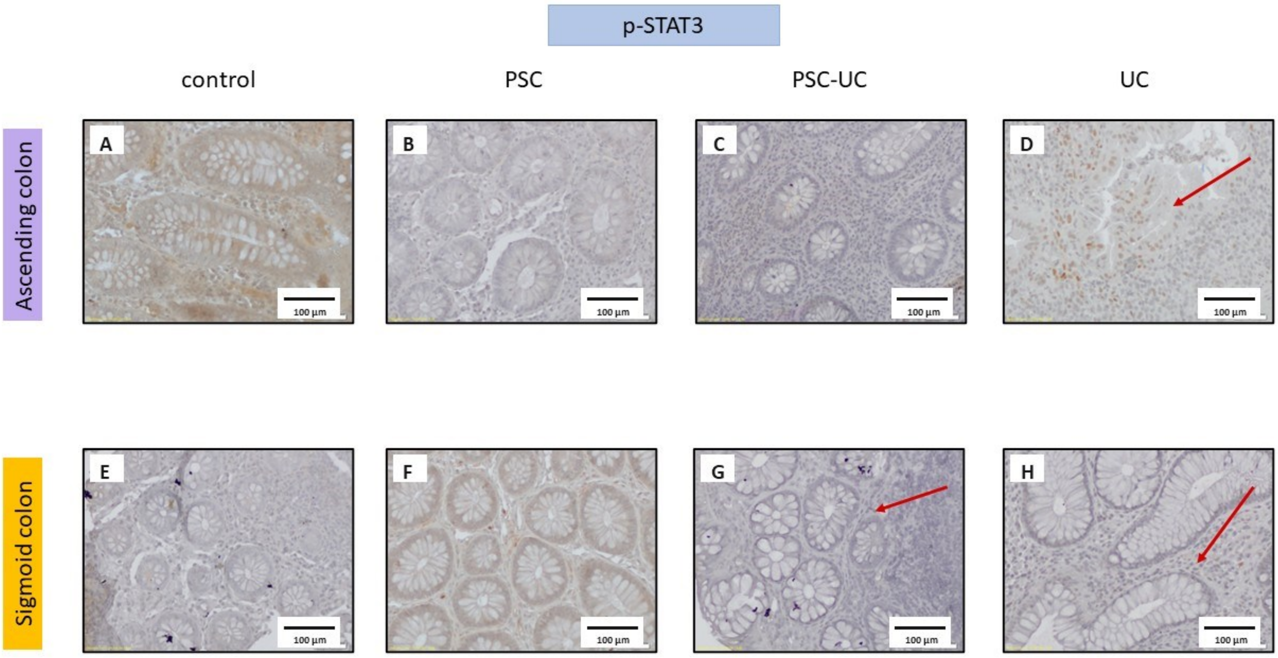

Figure S6. Representative immunohistochemical localization of phosphorylated STAT3 (p-STAT3) in the ascending and sigmoid colon. Representative sections from control subjects and patients with primary sclerosing cholangitis (PSC), primary sclerosing cholangitis-associated ulcerative colitis (PSC-UC), and ulcerative colitis (UC) are shown. (**A–D**) Ascending colon: Control, PSC, PSC-UC, and UC, respectively. (**E–H**) Sigmoid colon: Control, PSC, PSC-UC, and UC, respectively. p-STAT3 immunoreactivity is predominantly nuclear and is observed mainly in inflammatory cells within the lamina propria, whereas epithelial nuclear staining is weak or absent. Images are presented as a qualitative histological illustration of p-STAT3 localization; no quantitative IHC scoring or between-group statistical comparisons were performed. Scale bar = 100 µm.

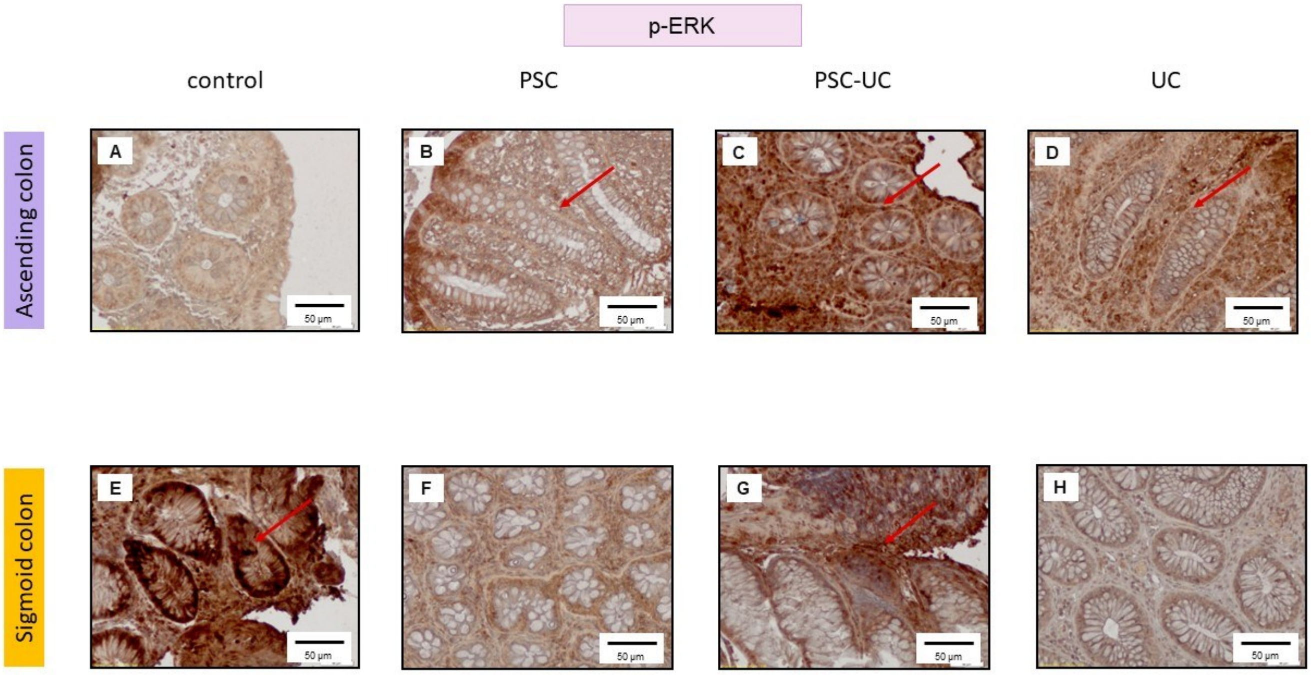

Figure S7. Representative immunohistochemical localization of phosphorylated ERK1/2 (p-ERK1/2) in the ascending and sigmoid colon. Representative sections from control subjects and patients with primary sclerosing cholangitis (PSC), primary sclerosing cholangitis-associated ulcerative colitis (PSC-UC), and ulcerative colitis (UC) are shown. (**A–D**) Ascending colon: Control, PSC, PSC-UC, and UC, respectively. (**E–H**) Sigmoid colon: Control, PSC, PSC-UC, and UC, respectively. p-ERK immunoreactivity is detected within the colonic mucosa, involving both epithelial and lamina propria compartments, with heterogeneous distribution and staining intensity among representative specimens and intestinal regions. Images are presented as a qualitative histological illustration of p-ERK localization; no quantitative IHC scoring or between-group statistical comparisons were performed. Scale bar = 100 μm.

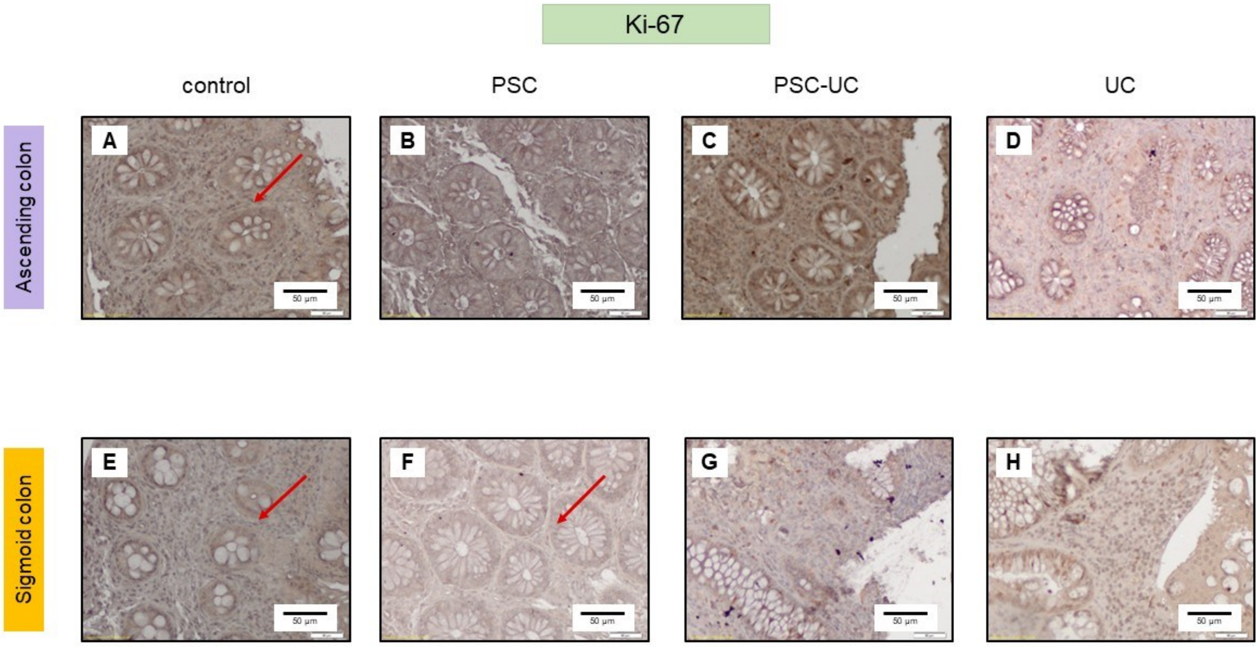

Figure S8. Representative immunohistochemical localization of Ki-67 in the ascending and sigmoid colon. Representative sections from control subjects and patients with primary sclerosing cholangitis (PSC), primary sclerosing cholangitis-associated ulcerative colitis (PSC-UC), and ulcerative colitis (UC) are shown. (**A–D**) Ascending colon: Control, PSC, PSC-UC, and UC, respectively. (**E–H**) Sigmoid colon: Control, PSC, PSC-UC, and UC, respectively. Ki-67 shows nuclear immunoreactivity within colonic epithelial cells, providing spatial visualization of epithelial proliferative activity. The distribution and apparent abundance of Ki-67-positive epithelial cells vary among representative specimens. Images are presented as a qualitative histological illustration of Ki-67 localization; no quantitative IHC scoring or between-group statistical comparisons were performed. Scale bar = 100 µm.

Table S1. Characteristics of the study cohorts.

A. Serum cohort

Parameter	*Control (n = 22)*	*PSC (n = 12)*	*PSC-UC (n = 18)*	*UC (n = 10)*
Female/Male, n	12/10	4/8	2/16	5/5
Age (years)	N/A	33 ± 4	33 ± 3	39 ± 4
Disease duration (years)	N/A	5.83 ± 0.75	9.71 ± 1.43	7.75 ± 2.22
CRP (mg/L)	N/A	N/A	N/A	28.29 ± 12.00
UC disease activity, active/inactive, n	N/A	N/A	18/0	10/0
Liver cirrhosis, Yes/No, n	N/A	3/9	7/11	N/A
Endoscopic activity, Mayo 1/2/3, n	N/A	N/A	N/A	3/3/4
Disease extent at diagnosis, pancolitis/non-pancolitis/not reported, n	N/A	N/A	N/A	2/6/2
5-ASA treatment, n/N	N/A	N/A	18/18	10/10
5-ASA dose, g/day	N/A	N/A	2-3	2-3
History of colorectal neoplasia, n/N	N/A	N/A	0/18	0/10
Hemoglobin (g/dL)	N/A	13.55 ± 0.29	17.16 ± 3.40	N/A
Bilirubin (mg/dL)	N/A	2.89 ± 0.91	2.83 ± 1.00	0.31 ± 0.04
ALP (U/L)	N/A	410.20 ± 103.71	365.94 ± 53.39	74.44 ± 9.33
ALT (U/L)	N/A	160.55 ± 35.01	138.00 ± 15.28	26.78 ± 6.32
AST (U/L)	N/A	121.36 ± 25.32	77.38 ± 9.61	25.89 ± 6.66
GGT (U/L)	N/A	489.30 ± 97.78	346.19 ± 70.15	26.00 ± 4.68
Total cholesterol (mg/dL)	N/A	234.67 ± 24.64	238.18 ± 20.76	N/A
Triglycerides (mg/dL)	N/A	115.20 ± 18.13	104.60 ± 20.46	N/A
DHEAS (μg/dL)	N/A	298.67 ± 149.86	148.76 ± 35.04	N/A

Note: UC activity status was based on contemporaneous clinical/endoscopic documentation; standardized Mayo endoscopic scores were available for the conventional UC group but not consistently for the PSC-UC group.

B. Sigmoid colon RNA cohort

Parameter	*Control (n = 13)*	*PSC (n = 12)*	*PSC-UC (n = 12)*	*UC (n = 10)*
Female/Male	5/8	2/10	3/9	8/2
Age (years)	N/A	31 ± 4	33 ± 3	43 ± 17
Hemoglobin (g/dL)	N/A	13.58 ± 0.93	13.60 ± 1.20	N/A
Bilirubin (mg/dL)	N/A	1.02 ± 0.30	2.84 ± 1.44	0.4 ± 0.2
ALP (U/L)	N/A	147.50 ± 24.09	425.17 ± 88.26	80 ± 24
ALT (U/L)	N/A	184.63 ± 97.42	177.82 ± 40.11	15 ± 8.4
AST (U/L)	N/A	118.13 ± 68.03	96.73 ± 22.22	N/A
GGT (U/L)	N/A	230.50 ± 41.93	447.27 ± 120.55	17 ± 13.3
Total cholesterol (mg/dL)	N/A	180.13 ± 21.84	212.56 ± 21.41	N/A
Triglycerides (mg/dL)	N/A	85.43 ± 7.39	116.44 ± 20.37	N/A
DHEAS (μg/dL)	N/A	220.00 ± 71.74	106.01 ± 32.83	N/A

C. Sigmoid colon Western blot cohort

Parameter	*Control (n = 12)*	*PSC (n = 3)*	*PSC-UC (n = 4)*	*UC (n = 11)*
Female/Male	5/7	1/2	1/3	8/2
Age (years)	N/A	39 ± 11	33 ± 3	43 ± 17
Hemoglobin (g/dL)	N/A	13.65 ± 2.05	N/A	N/A
Bilirubin (mg/dL)	N/A	0.98 ± 0.60	0.64 ± 0.23	0.4 ± 0.2
ALP (U/L)	N/A	82.50 ± 3.50	541.75 ± 180.39	80 ± 24
ALT (U/L)	N/A	41.00 ± 20.00	220.25 ± 111.72	15 ± 8.4
AST (U/L)	N/A	36.50 ± 13.50	102.25 ± 52.84	N/A
GGT (U/L)	N/A	204.00 ± 129.00	341.00 ± 93.12	17 ± 13.3
Total cholesterol (mg/dL)	N/A	170.00 ± 67.00	164.50 ± 11.50	N/A
Triglycerides (mg/dL)	N/A	93.00 ± 1.00	92.50 ± 20.50	N/A
DHEAS (µg/dL)	N/A	155.50 ± 48.50	78.30 ± 37.39	N/A

D. Ascending colon RNA cohort

Parameter	*Control (n = 12)*	*PSC (n = 12)*	*PSC-UC (n = 11)*	*UC (n = 10)*
Female/Male	4/8	2/10	3/8	8/2
Age (years)	N/A	31 ± 4	35 ± 5	43 ± 17
Hemoglobin (g/dL)	N/A	13.58 ± 0.93	13.6 ± 1.20	N/A
Bilirubin (mg/dL)	N/A	1.02 ± 0.30	3.07 ± 1.58	0.4 ± 0.2
ALP (U/L)	N/A	147.50 ± 24.09	458.64 ± 89.46	80 ± 24
ALT (U/L)	N/A	184.63 ± 97.42	187.40 ± 43.06	15 ± 8.4
AST (U/L)	N/A	118.13 ± 68.03	101.90 ± 23.88	N/A
GGT (U/L)	N/A	230.50 ± 41.93	467.00 ± 131.48	17 ± 13.3
Total cholesterol (mg/dL)	N/A	180.13 ± 21.84	204.88 ± 22.66	N/A
Triglycerides (mg/dL)	N/A	85.43 ± 7.38	119.63 ± 22.81	N/A
DHEAS (µg/dL)	N/A	220.00 ± 71.74	120.87 ± 33.81	N/A

Note: Because the availability of biological material differed among patients, separate cohorts were used for serum, RNA and Western blot analyses; therefore, sample sizes vary between subgroups.

E. Ascending colon Western blot cohort

Parameter	*Control (n = 11)*	*PSC (n = 6)*	*PSC-UC (n = 7)*	*UC (n = 10)*
Female/Male	4/7	1/5	2/5	8/2
Age (years)	N/A	32 ± 5	34 ± 6	43 ± 17
Hemoglobin (g/dL)	N/A	12.35 ± 0.45	14.60 ± 1.19	N/A
Bilirubin (mg/dL)	N/A	1.25 ± 0.56	3.30 ± 2.43	0.4 ± 0.2
ALP (U/L)	N/A	177.00 ± 40.75	464.71 ± 156.67	80 ± 24
ALT (U/L)	N/A	275.75 ± 194.12	200.71 ± 61.44	15 ± 8.4
AST (U/L)	N/A	176.75 ± 138.15	88.29 ± 29.26	N/A
GGT (U/L)	N/A	262.00 ± 68.42	464.14 ± 156.67	17 ± 13.3
Total cholesterol (mg/dL)	N/A	184.75 ± 37.55	227.60 ± 32.56	N/A
Triglycerides (mg/dL)	N/A	88.00 ± 17.67	138.00 ± 35.01	N/A
DHEAS (µg/dL)	N/A	245.80 ± 99.96	74.48 ± 26.71	N/A

Table S2. Receiver operating characteristic (ROC) analysis of the most informative molecular markers identified by Random Forest for discrimination between PSC-UC and UC in the ascending and sigmoid colon.

Tissue	Marker	AUC (95% CI)	Cut-off	Sensitivity (%)	Specificity (%)
**Ascending colon**	CYLD mRNA	0.549 (0.338–0.760)	0.785	40.0	88.9
	PTEN mRNA	0.443 (0.226–0.660)	1.240	65.5	50.0
	**miR-181a**	**0.806 (0.651–0.960)**	**1.310**	**66.7**	**100**
	p-AKT protein	0.709 (0.501–0.917)	1.845	58.8	100
**Sigmoid colon**	**p65 mRNA**	**0.668 (0.500–0.836)**	**0.695**	**58.6**	**100**
	miR-21	0.617 (0.331–0.902)	0.640	80.0	57.1
	miR-181a	0.612 (0.429–0.794)	1.276	53.8	100
	PTEN mRNA	0.447 (0.255–0.638)	1.055	40.0	80.0

Table S3. Discriminatory performance of individual circulating miRNAs and the exploratory multivariable serum miRNA profile for distinguishing PSC-UC from UC based on receiver operating characteristic (ROC) curve analysis. The optimal cut-off value was determined using Youden's index.

Marker	*N*	*AUC (95% CI)*	*Cut-off*	*Sensitivity (%)*	*Specificity (%)*	*Direction*
miR-155	24	0.797 (0.613–0.981)	0.85	50.0	100	<
miR-21	26	0.647 (0.429–0.865)	0.865	58.8	77.8	<
miR-181a	26	0.582 (0.350–0.814)	1.96	29.4	100	<
miR-181b	26	0.547 (0.312–0.782)	2.00	50.0	90.0	<
Multivariable serum miRNA profile	21	1.000 (1.000–1.000)	0.500	100	100	—

Note: The multivariable serum miRNA profile was derived from logistic-regression predicted probabilities in the complete-case subset (N = 21; PSC-UC, n = 14; UC, n = 7), with PSC-UC defined as the positive class. The Youden-optimal predicted-probability cut-off was 0.500, corresponding to a sensitivity of 100%, specificity of 100%, and Youden's J of 1.000. Paired DeLong comparisons were performed in the same 21 subjects; therefore, the marker AUCs used for these comparisons differ from the full marker-specific AUCs reported above.

Table S4. Paired DeLong comparisons of the multivariable serum miRNA profile with individual circulating miRNAs in the common complete-case cohort (N = 21).

Comparison	AUC Multivariable Profile (95% CI)	AUC Marker (95% CI)	DeLong Z	p value
Multivariable profile vs miR-155	1.000 (1.000–1.000)	0.816 (0.626–1.000)	1.894	0.05824
Multivariable profile vs miR-21	1.000 (1.000–1.000)	0.673 (0.432–0.915)	2.653	0.007977
Multivariable profile vs miR-181a	1.000 (1.000–1.000)	0.592 (0.336–0.848)	3.124	0.001786
Multivariable profile vs miR-181b	1.000 (1.000–1.000)	0.423 (0.163–0.684)	Not reported*	Not reported*

* Multivariable miRNA profile vs miR-181b: R issued the warning "DeLong's test should not be applied to ROC curves with a different direction." Because the multivariable miRNA profile and miR-181b ROC curves had different directions, the DeLong Z statistic and p value were not reported or interpreted.

Table S5. Internal performance analysis of the multivariable serum miRNA profile derived using logistic regression in the complete-case cohort (N = 21; PSC-UC, n = 14; UC, n = 7)

Analysis	*Procedure*	*AUC*	*Variability / 95% CI*	*Model refitted?*
Apparent ROC	Fit and evaluated in the same N = 21 sample	1.000	95% CI: 1.000–1.000	Single original fit
Bootstrap	2000 resamples of predictions from the originally fitted model	1.000	95% CI: 1.000–1.000	No
Cross-validation, mean	Repeated stratified five-fold CV, 100 repetitions	0.951	SD = 0.050	Yes, in every training fold
Cross-validation, pooled OOF	Pooled out-of-fold predicted probabilities across 100 repeated stratified five-fold CV runs	0.933	Reported SD = 0.068	Yes, in every training fold

Note: Bootstrap interpretation. Because the logistic-regression model was not refitted within each bootstrap sample, the bootstrap result is not an optimism-corrected AUC; it reflects resampling of performance from the already fitted model and should not be used as evidence that overfitting was removed.

Cross-validation procedure. Repeated stratified five-fold cross-validation was used, preserving the PSC-UC:UC class proportions, with 100 independent repetitions. Within each fold, the logistic-regression model was refitted on the corresponding training set (four-fifths of the observations) and evaluated on the held-out fold. Across repetitions, the mean of the repetition-level mean fold AUCs was 0.951 (SD = 0.050), whereas the mean AUC calculated from pooled out-of-fold predictions within each repetition was 0.933 (SD = 0.068).

Logistic-regression fitting. R reported “glm.fit: algorithm did not converge” and “glm.fit: fitted probabilities numerically 0 or 1 occurred”. These warnings indicate numerical instability compatible with complete or quasi-complete separation. Thus, absence of separation cannot be confirmed and the apparent AUC = 1.000 requires cautious interpretation.
